# Studies on B Cells in the Fruit-Eating Black Flying Fox (*Pteropus alecto)*

**DOI:** 10.3389/fimmu.2019.00489

**Published:** 2019-03-14

**Authors:** Pravin Periasamy, Paul E. Hutchinson, Jinmiao Chen, Isabelle Bonne, Shahana Shereene Shahul Hameed, Pavithra Selvam, Ying Ying Hey, Katja Fink, Aaron T. Irving, Charles-Antoine Dutertre, Michelle Baker, Gary Crameri, Lin-Fa Wang, Sylvie Alonso

**Affiliations:** ^1^Department of Microbiology and Immunology, Yong Loo Lin School of Medicine, National University of Singapore, Singapore, Singapore; ^2^Immunology Programme, Life Sciences Institute, National University of Singapore, Singapore, Singapore; ^3^Singapore Immunology Network (SIgN), Agency for Science, Technology and Research (A^*^STAR), Singapore, Singapore; ^4^Programme in Emerging Infectious Disease, Duke-NUS Medical School, Singapore, Singapore; ^5^CSIRO, Livestock Industries, Australian Animal Health Laboratory, Geelong, VIC, Australia; ^6^Crameri Research Consulting, Geelong, VIC, Australia

**Keywords:** bat, adaptive immunity, *Pteropus alecto*, *Eonycteris spelaea*, cross-reactive antibodies

## Abstract

The ability of bats to act as reservoir for viruses that are highly pathogenic to humans suggests unique properties and functional characteristics of their immune system. However, the lack of bat specific reagents, in particular antibodies, has limited our knowledge of bat's immunity. Here, we report a panel of cross-reactive antibodies against MHC-II, NK1.1, CD3, CD21, CD27, and immunoglobulin (Ig), that allows flow cytometry analysis of B, T and NK cell populations in two different fruit-eating bat species namely, *Pteropus alecto* and *E. spelaea*. Results confirmed predominance of T cells in the spleen and blood of bats, as previously reported by us. However, the percentages of B cells in bone marrow and NK cells in spleen varied greatly between wild caught *P. alecto* bats and *E. spelaea* colony bats, which may reflect inherent differences of their immune system or different immune status. Other features of bat B cells were investigated. A significant increase in sIg^+^ B cell population was observed in the spleen and blood from LPS-injected bats but not from poly I:C-injected bats, supporting T-independent polyclonal B cell activation by LPS. Furthermore, using an *in vitro* calcium release assay, *P. alecto* B cells exhibited significant calcium release upon cross-linking of their B cell receptor. Together, this work contributes to improve our knowledge of bat adaptive immunity in particular B cells.

## Introduction

Classified in the order Chiroptera and the only flying mammals (1), bats have long been of great interest to the scientific community for their unique features, including their long lifespan that is counter to the established relationship between body mass and longevity (2, 3), their low incidence of cancer (4) and their ability to harbor deadly pathogens without signs of disease. Indeed, bats have been shown to act as reservoirs for viruses that otherwise result in fatal outcomes in humans, including rabies virus, ebola virus, Nipah virus, Hendra virus, and MERS-CoV virus (5). Each year, new viruses are isolated from bats (6, 7). This suggests that bats have co-evolved with these viruses and are able to control and/or contain them (8). However, the mechanisms involved remain to be fully elucidated.

Bats have high metabolic rate during flight and low metabolic rate in the day during their shallow torpor. This has been thought to play a role in their ability to cope with infections as studies in mice with high metabolic rates have suggested enhanced immune response, as evidenced by increased IgM production, leukocyte proliferation and mass of lymphatic organs (9). In addition, it has been suggested that elevated body temperature resulting from high metabolic rate may help bats control infections (10). On the other hand, other studies have suggested that bats' low metabolism during the day could lead to immune response suppression thereby allowing better co-adaptation with viruses (11, 12).

Alternatively, or in addition, bats' ability to host pathogens may stem from their immune system. Study of the bat immune system has been hampered by the lack of tools and resources available. The difficulty to establish bat colonies similar to mouse colonies has forced scientists to work with wild caught bats, which inevitably results in significant bat-to-bat variations, making data interpretation difficult. Furthermore, limited tools including bat cell lines, and bat reagents (bat molecules, specific antibodies, etc.) are available. Therefore, most of the knowledge accumulated so far has derived from genome and transcriptome analyses including full genome sequencing, RNA-Seq and real time PCR (13).

Innate immunity in bats has been studied in several species mostly at the genome and transcriptome levels, and has shown notable similarity with other mammals (1, 14). Common innate immune defense mechanisms are found in bats such as Toll-like receptors (TLRs) (15, 16). Various types of interferons (IFN) have also been described in bats. The type I IFN response to viral infections was found to be similar to that of other mammals (17, 18). Interestingly, high expression level of type I IFN “IFNω” was detected in bats, which is not common in other mammals (8). Type II IFN or IFNγ was found to affect the replication of Hendra virus in black flying fox *Pteropus alecto* cells and of Sendai virus in cells from Brazilian free-tail bat *Tadarida brasiliensis* (19) whereas Type III IFN was reported to be produced in bats injected with Tioman virus (20). These findings suggest that bats express diverse IFN pathways that could play an important role in controlling viral infections.

The adaptive immune system in bats has been less studied, partly due to the lack of specific tools in particular the absence of bat-specific antibodies (21) and the striking poor cross-reactivity of antibodies that recognize lymphocyte cell surface markers in other mammals (22). Genomic and transcriptomic approaches have identified the presence of T cell subsets (23). In a previous study, using cross-reactive antibodies specific to transcription factors, we were able to identify CD4^+^ and CD8^+^ T cell populations, and reported the unusual dominant proportion of CD8^+^ T cells in *P. alecto* secondary lymphoid organs, which may suggest that bat's adaptive immune system is geared toward controlling intracellular pathogens, typically viruses (22). Other studies have described the detection of high titres of circulating IgG antibodies in bats (24). Transcripts of IgM, IgE, IgA, and several IgG subclasses were also detected. Of interest, IgG antibodies were found in abundance in maternal lacteal secretions as opposed to IgA predominance in other mammals, which may have important implications for the transfer of maternal immunity and protection in the bat progeny against systemic infections (25, 26). Presence of IgD in some microbats but not in megabats has also been reported, illustrating a significant variability in antibody species across bats species (8).

Here, we report for the first time a set of cross-reactive antibodies that can be used to study B, T and NK cell populations in the fruit-eating bats *P. alecto* and *E. splelaea*. We found that proportions of these adaptive immune cell subsets in blood, spleen and bone marrow differed substantially from other mammals. A calcium release assay was also optimized upon cross-linking of the B cell receptor with a specific antibody. Finally, we found that LPS-treated bats displayed increased B cell subset only 5 h post-treatment suggesting an unusually fast response to this polyclonal B cell activator.

## Materials and Methods

All the *ex vivo* biological experiments described were conducted in a Biosafety Level 2 containment facility and were approved by the institutional biosafety committee of National University of Singapore.

### Animals

*Pteropus alecto* bats used in this study were caught in Queensland (Australia) and transported to the Australian Animal Health Laboratory (AAHL), CSIRO (Victoria, Australia). *E. spelaea* were caught in Singapore and housed for a period of 6 months at the Sing Health Experimental Center. Peripheral blood mononuclear cells (PBMC), bone marrow and spleen were harvested and single cell suspensions were prepared in RPMI containing 10% dimethylsulfoxide (DMSO) and 90% fetal bovine serum (FBS). The vials were then slowly frozen overnight at −80°C in a Styrofoam “sandwich” box, and then placed in liquid nitrogen tank for long term storage and until further analysis.

Only bats testing negative by qPCR for Hendra virus (HeV) and lyssavirus were included in the study. The procedures performed on *P. alecto* bats were approved by the Queensland Animal Science Precinct (QASP)/University of Queensland (AEC #SVS/073/16/USGMS). *Eonycteris spelaea* bats handling and processing work were conducted in accordance with approved guidelines, methods and permits from Duke-NUS Medical School and SingHealth Experimental Medicine Centre (2015/SHS/1088).

### LPS and Poly I:C Treatments

Wild caught *P. alecto* bats (*n* = 3 per group) were injected intraperitoneally with 2 mg/kg of either lipopolysaccharide (LPS) purified from *E. coli* 055:B5 (Invivogen) or high molecular weight (HMW) Poly I:C (Invivogen), or left untreated. Five hours post-injection, the animals were euthanized and their spleen, bone marrow, lymph nodes, and PBMC were harvested and single cell suspensions were prepared and stored in liquid nitrogen until further analysis.

### Sample Processing for Flow Cytometry Analysis

Single cell suspensions from spleen, mediastinal lymph nodes, bone marrow or PBMC were stained with Fixable Viability Dye e780 (eBioscience) for 20 min at 4°C, then 10% FBS was added and cells were incubated for another 10 min. For staining of surface markers, the cells were incubated first with fluorescent-conjugated primary antibodies including anti-mouse I-A/I-E MHC class II (clone 2G9, BD), anti-mouse CD11b (clone M1/70, eBioscience), anti-human CD21 (clone B-ly-4, eBioscience), anti-mouse CD27 (clone LG.7F9, eBioscience), and anti-mouse NK1.1 (clone PK136, Biolegend). Incubation with non-conjugated polyclonal goat anti-bat Ig (Novus Biologicals, NB7237), for 30 min at 4°C, was followed by incubation with fluorescently labeled anti-goat IgG secondary antibody (ThermoFisher) for 30 min at 4°C. For staining of intracellular proteins including Ig and cytoplasmic domain of CD3 (clone CD3-12, Biorad), the cells were first fixed and permeabilized using the Fix/Perm Foxp3-staining kit (eBioscience) for 30 min at 4°C. Staining was performed at room temperature (RT) in the dark. Each antibody was titrated to determine the optimal concentration for use. Antibody dilution was made in FACS buffer (PBS with 5% Fetal Bovine Serum). For every sample, a fluorescent minus one (FMO) control was set up, defined as the sample incubated with all the antibodies minus one. Typically, 100,000 cells were acquired. Samples were analyzed using LSR Fortessa X-20 (BD) and Flowjo software Version 10 (Flowjo LLC, Ashland, Oregon, USA).

### PrimeFlow-Fluorescence *in-situ* Hybridization (Flow-FISH)

The FlowRNA I Assay kit (Affymetrix eBioscience) was used according to the manufacturer's instructions. Briefly, one million cells per sample were first stained with a live fixable viability dye (eBioscience), then fixed and permeabilized according to the manufacturer's instructions. Cells were then incubated with anti-CD3 antibody for 30 min at RT, followed by *in-situ* hybridization with the Alexa Fluor 647-labeled CD19 mRNA oligo probes (1 μM final concentration for each oligo). Analysis was done using LSRFortessa X-20 (BD) and Flowjo software Version 10.

### Calcium Flux Assay

Frozen single cell suspensions prepared from bone marrow (BM), spleen (SPL), PBMC and lymph nodes (LN) were thawed. Cells were incubated with a calcium sensor dye, Indo-1 AM (eBioscience, USA) for 1 h at 37°C + 5% CO_2_. Unbound Indo-1 has a peak emission at 475 nm. Upon binding calcium, the peak emission shifts down to 410 nm. Indo-1 labeled cells were then surfaced-stained for mouse MHC-II and CD11b, and goat anti-bat Ig. Samples (four technical replicates of each) were incubated at 37°C for 5 min and 100,000 cells were acquired in a flow cytometer (BD X-20 analyser) for approximately 30 s to determine baseline calcium concentration in cells. Samples were removed and donkey anti-goat 2nd antibody was added to the samples. The absorbance ratio measured at the two emission wavelengths was measured before and after adding IgG.

### Scanning Electron Microscopy (SEM)

Approximately 5 × 10^6^ B cells defined as sIg^+^ MHC II^+^ CD11b^−^ were sorted using the using the Sony Sy3200 cell sorter into tubes. Propidium iodide (PI) was used to exclude dead cells and purity of the collected cells was checked by running the sample back in the sorter. Sorted B cells were applied to a glass coverslip, and fixed with 0.25% glutaraldehyde in 0.1 M Sorensen buffer (pH 7.2) for 1 h, then washed three times for 5 min (each time) in PBS, post-fixed for 1 h in 1% osmium tetroxide and finally rinsed with distilled water. Cells were dehydrated through a graded series of ethanol (25, 50, 75, 95, and 100%) followed by critical point drying with CO_2_. Dried specimens were sputter coated with gold, with a BALTEC SCD005 evaporator and were examined and photographed with a JEOL JSM 6701F field emission scanning electron microscope operating at 10 kV.

### Statistical Analyses

Students *t*-test was performed on the control, LPS treated and Poly I:C treated bat groups and on B, T and myeloid cell subsets in the Calcium flux assay using Graphpad Prism Software (Graphpad Software, Inc., San Diego, United States). Mean and standard deviation was calculated for each dataset.

## Results

### Screening of Cross-Reactive Antibodies for *P. alecto* B Cells Identification

Eighty-two commercially available antibodies that recognize lymphocyte cell surface markers from other mammal species were screened by flow cytometry on single cell suspensions prepared from *P. alecto* spleen and bone marrow (Table S1). In addition, a commercially available polyclonal goat anti-bat IgG antibody was included in the panel. However, although this anti-bat IgG polyclonal antibody is claimed to be specific to bat IgG, it is conceivable that it may also bind to a certain extent to the conserved kappa and lambda light chains of other Ig classes. It is therefore possible that in addition to sIgG^+^ B cells, this antibody allows detect for other B cell subsets including those that express IgM or IgD at their surface. Therefore, to account for this possibility, cells that are stained with this polyclonal antibody will be denoted Ig^+^.

The choice of these 83 antibodies was based on their ability to help identify B cells, either directly through binding to B cell surface markers or indirectly through binding to non-B cells, including T cells, NK cells, myeloid cells, and hematopoietic stem cells (Table 1). Single, live cells from the leukocyte population were gated and analyzed by flow cytometry. A shift of a defined cell subset compared to unstained sample was clearly observed for 15 of the antibodies tested (Tables 1, 2). However, some of these antibodies (e.g., CD4 RM44) were excluded due to non-specific binding, as evidenced by very high percentages of positive cells (in the range of 80–90%) (Figure S1). In addition, others did not give a clear and distinct population upon flow cytometry analysis and have thus been excluded as well (Figure S1). Eventually, seven antibodies were deemed bona fide cross-reactive antibodies for the study of B cell populations (Table 2). They include antibodies that recognize B cell surface markers (sIg, CD21, CD27, MHC-II) as well as antibodies that recognize non-B cell markers (CD11b, NK.1.1, intracellular CD3).

**Table 1:** List of antibodies tested by FACS.

**Target immune cell subset**	**Total antibodies tested**	**Antibodies with a signal above background**	**True cross-reactive antibodies**
T cells	22	2	1
B cells	15	6	3
NK cells	5	2	1
Myeloid cells	7	2	2
Other cells	34	3	-
**Total**	**83**	**15**	**7**

**Table 2:** Detection of immune cell subsets in bat bone marrow and spleen using cross-reactive antibodies.

**Antibody tested against**	**Bone marrow % of population (+)**	**Spleen % of population (+)**	**Species**
CD11b	50.70	36.30	Anti-Mouse
Ig	37.80	39.40	Anti-Bat
CD21	9.76	27.10	Anti-Human
CD27	19.00	14.30	Anti-Human
NK1.1	17.00	30.30	Anti-Mouse
CD3	21.70	15.60	Anti-Human
MHC-II (I-A/I-E)	23.50	66.30	Anti-Mouse

The limited number of true cross-reactive antibodies that were identified in this study further supports our previous findings that surface markers of adaptive immune cells are not well conserved between bats and other mammals such as humans and rodents (22).

### Antibody Staining Strategy to Study B, T and NK Cell Populations in *P. alecto* Bats

**Figure 1:**
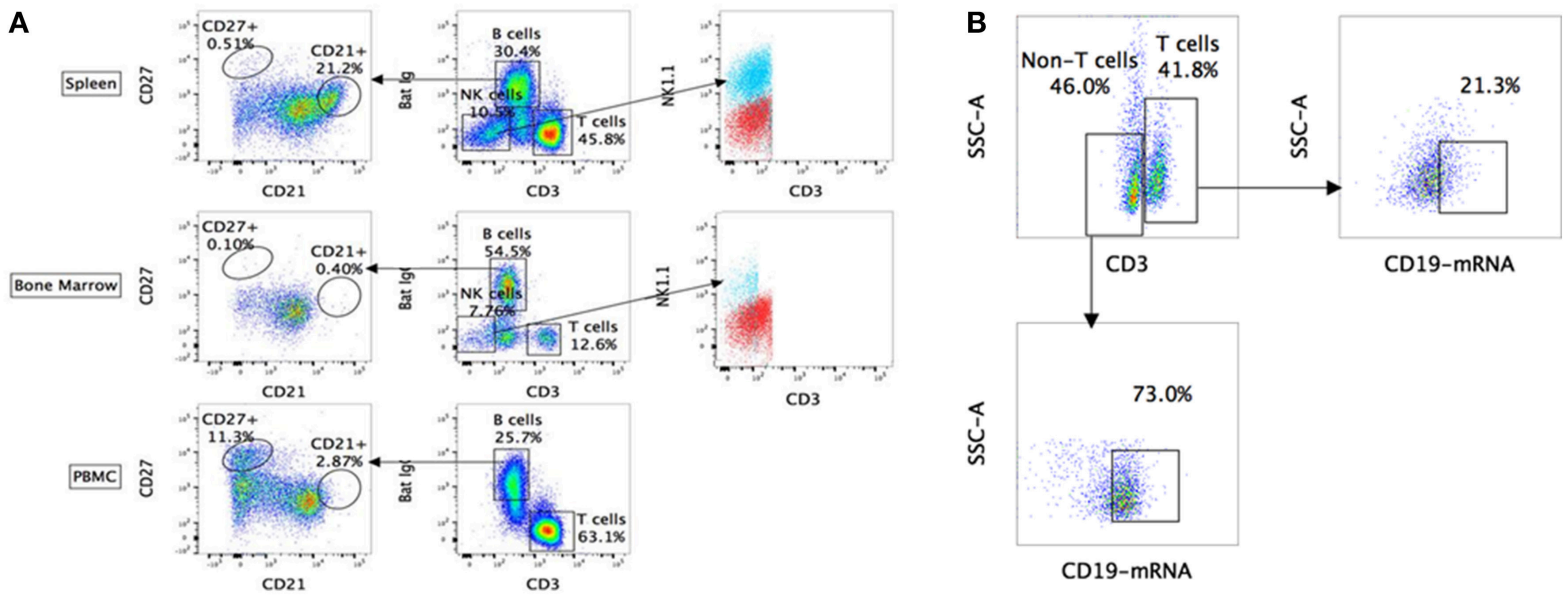
Flow cytometry analysis of *P. alecto* immune cells using cross-reactive antibodies and Flow-FISH. **(A)** *P. alecto* immune cells from spleen, bone marrow and peripheral blood mononuclear cell (PBMC) were analyzed by flow cytometry. CD3^+^ sIg^−^ cells were identified as T cells while CD3^−^ sIg^−^ cells as NK cells and CD3^+/−^ sIg^+^ as B cells. B cell population that was CD27^+^ was identified as memory cells and CD21^+^ as mature B cells. CD3^−^ sIg^−^ cells were further verified using NK1.1 antibody to confirm NK cell population. **(B)** Detection of B cell subsets by combined Flow-FISH and antibody staining. Bat splenocytes were stained with CD3 antibody followed by *in-situ* hybridization with probes specific for CD19_mRNA_. One data set is shown and is representative of 3 independent experiments performed with 3 different bat bone marrow.

**Figure 2:**
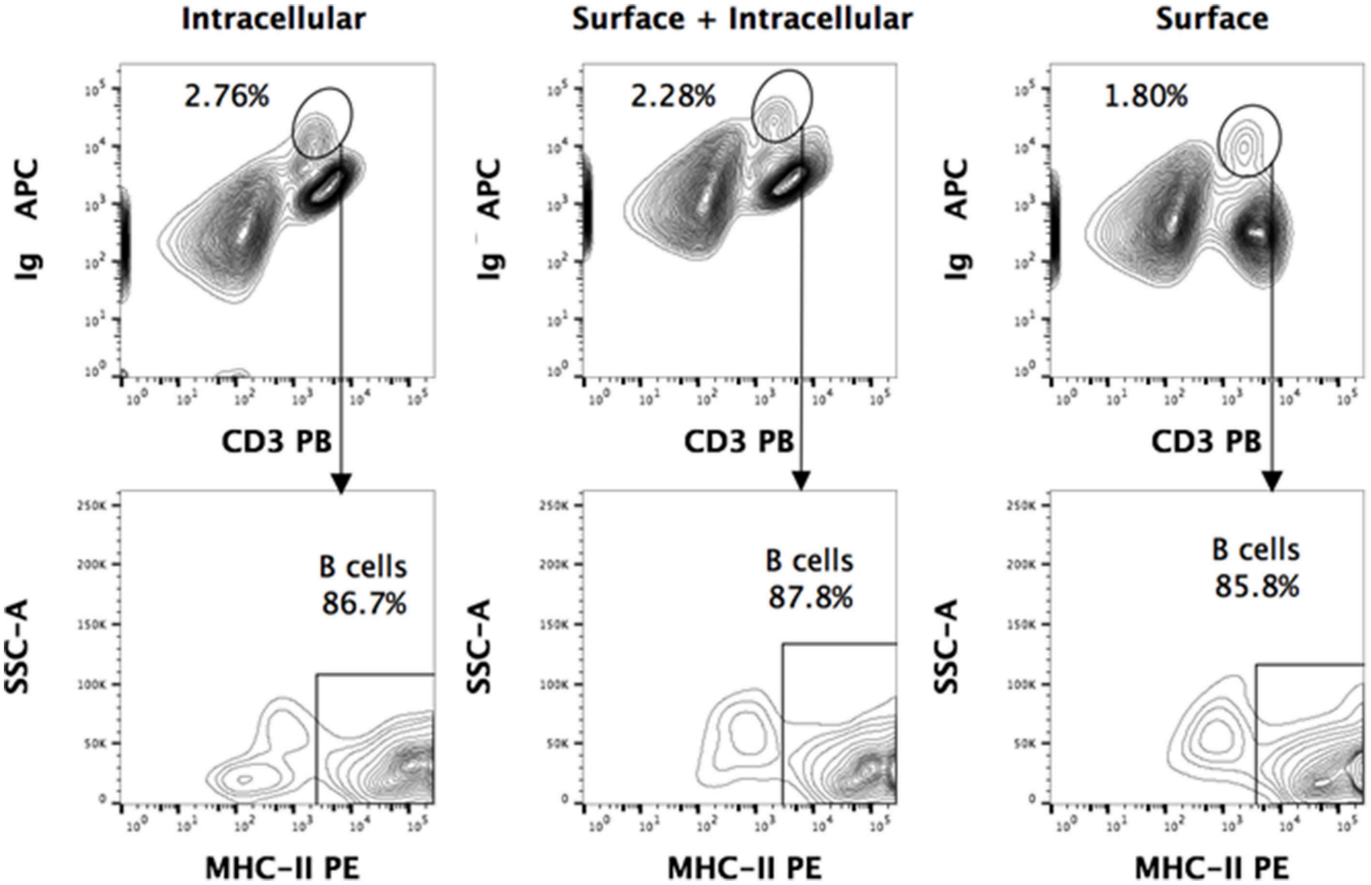
Intracellular and surface Ig staining of *P. alecto* splenocytes. Dead cells were excluded using Live/Dead eFluor 506 dye and live cells were gated for singlets using forward scatter height (FSC-H) and area (FSC-A). For all the staining approaches (surface only, intracellular only, or surface + intracellular), a distinct sIg^+^ CD3^+/−^ population is observed. Greater than 85% of this population is MHCII^+^. Comparable percentages of these cells were obtained with surface staining only vs. intracellular staining only, thus strongly suggesting that this population is truly a sIg^+^ B cell population.

**Figure 3:**
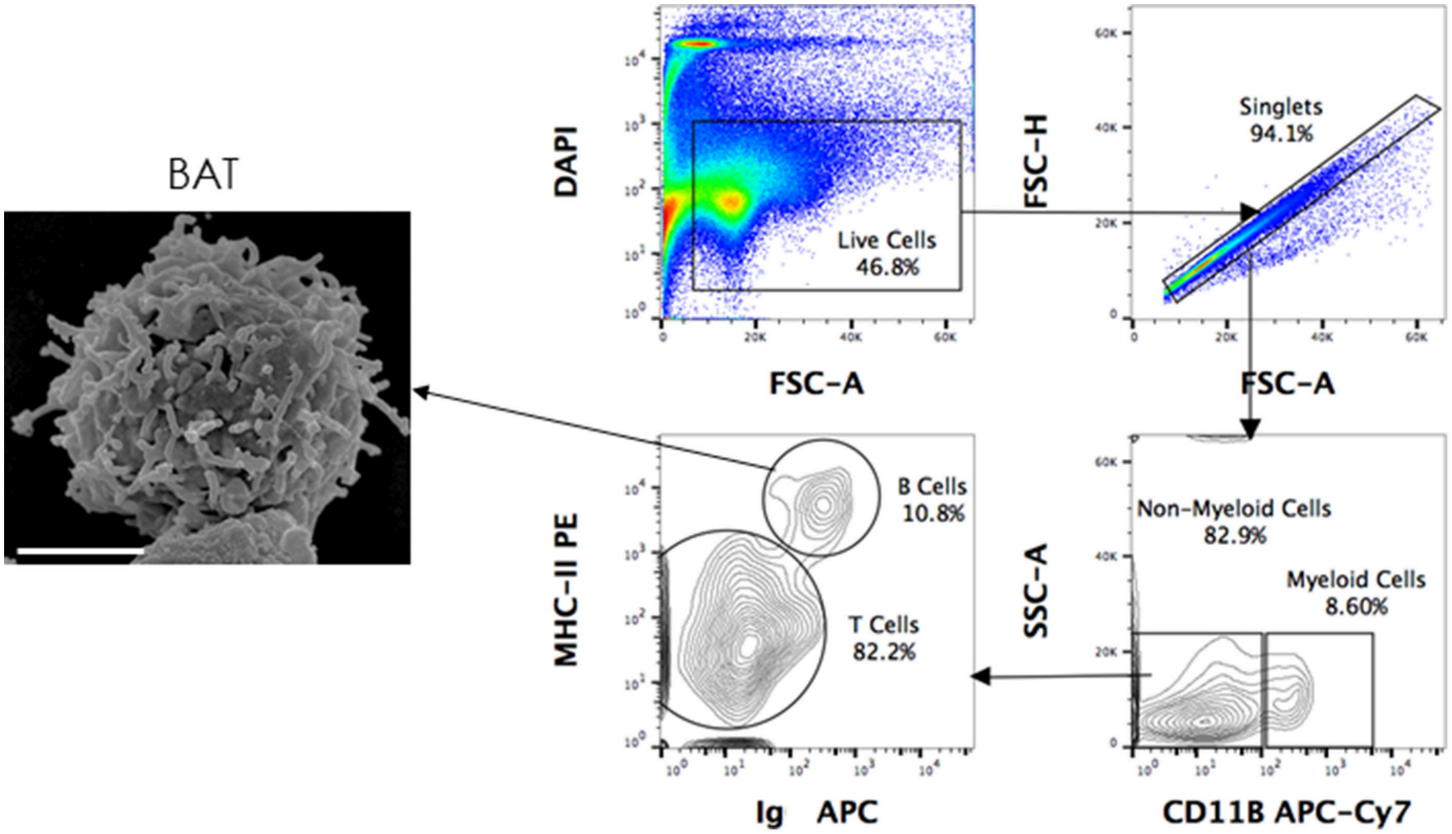
Scanning electron microscopy (SEM) of FACS sorted B cells. *P. alecto* splenocytes were surface-stained for CD11b, MHC-II and Ig antibodies and sorted as CD11b^−^ MHC-II^+^ sIg^+^ (B cells), CD11b^+^ (myeloid cells) and CD11b^−^ MHC-II^−^ sIg^−^ cells (T cells). Sorted B cells were processed for viewing under a scanning electron microscope. Scanned electron micrograph of sorted *Pteropus alecto* MHC-II^+^sIg^+^ B cells. Bar represents 2 μm.

Using the above-mentioned panel of cross-reactive antibodies, various adaptive immune cell populations including T cells (CD3^+^), NK cells (CD3^−^ sIg^−^ NK1.1^+^), and B cells (CD3^+/−^ sIg^+^) present in the bone marrow, spleen and PBMC of four wild caught *P. alecto* bats were analyzed by flow cytometry (Figure 1A). Among the B cells, two different subsets could be identified based on their CD27 and CD21 expression that may correspond to memory and mature B cells, respectively. However, due to the lack of an appropriate Fc block, it is plausible that antibodies could bind to Fc receptors (FcR) expressed at the surface of myeloid cells, thereby giving rise to false positive. To test this hypothesis, intracellular staining with anti-Ig antibody was performed, as well as a combination of surface and intracellular staining. The staining profiles obtained were comparable to that obtained when surface staining alone was performed, thus indicating that the cell population identified very likely consists of B cells, not myeloid cells (Figure 2). Furthermore, the CD3^+/−^ sIg^+^ B cell population was confirmed by the detection of CD19 mRNA by Prime Flow (Figure 1B). Finally, using cross-reactive antibodies against bat Ig, MHC-II and CD11b, CD11b^−^ MHC-II^+^ sIg^+^ cells were FACS sorted and examined under field scanning electron microscope (SEM). SEM images showed that these cells display a morphology that is similar to that of B cells from humans and mice in terms of size and gross appearance (Figure 3) (27), which further supports that our antibody staining strategy allows identification and study of the bat B cell populations.

### Analysis of Adaptive Immune Cell Populations in Bone Marrow, Spleen and PBMC From *P. alecto* Bats

**Figure 4:**
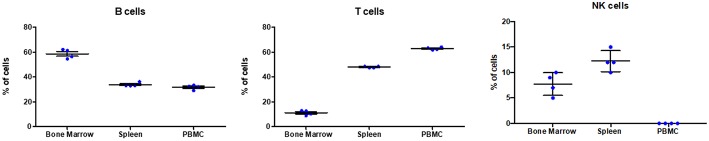
Analysis of B, T and NK cell populations in blood, spleen and bone marrow from *P. alecto*. *P. alecto* B, T and NK cell populations from spleen, bone marrow and PBMC were analyzed by flow cytometry as described in the legend of Figure 1A. The percentages of B, T and NK cell populations in the bone marrow, spleen and PBMC of four *P. alecto* bats are shown.

Based on the antibody staining strategy described above, the B, T, and NK cell populations were analyzed in the blood and various lymphoid organs from four wild caught *P. alecto* bats. All four bats displayed similar percentages of B, T and NK cell populations suggesting that this group of four animals was rather homogenous in term of immune status (Figure 4). Overall, higher percentages of T cells than B cells were found in the PBMC and spleen, whereas higher percentages of B cells than T cells were seen in the bone marrow (Figure 4). The percentages of NK cells in the four bats ranged between 10 and 16% in spleen and between 5 and 11% in bone marrow (Figure 4). Compared to mice that typically harbor 30–35% T cells and 45–50% B cells in their spleen, *P. alecto'*s spleen contains greater percentages of T cells and lower percentages of B cells (Table 3). Similarly, higher percentages of B cells compared to T cells are typically found in mice PBMC, whereas the reverse is seen in bat PBMC (Table 3). When compared to human PBMC, which typically consist of about 3–15% B cells and 40–60% T cells, the percentages of these two immune cell populations in *P. alecto* bats are much higher (Table 3).

**Table 3:** Comparison of B and T cell percentages in bat, murine and human tissues.

	**% Of cell population**					
**Cell type**	***P. alecto***		**Mice**		**Humans**	
	**PBMC**	**Spleen**	**PBMC**	**Spleen**	**PBMC**	**Spleen**
B cells	29–33	33–36	35–58	45–50	3–15	-
T cells	62–64	47–49	17–20	30–35	40–60	-

### Analysis of B Cell and T Cell Populations in *P. alecto* Bats Treated With LPS or Poly I:C

**Figure 5:**
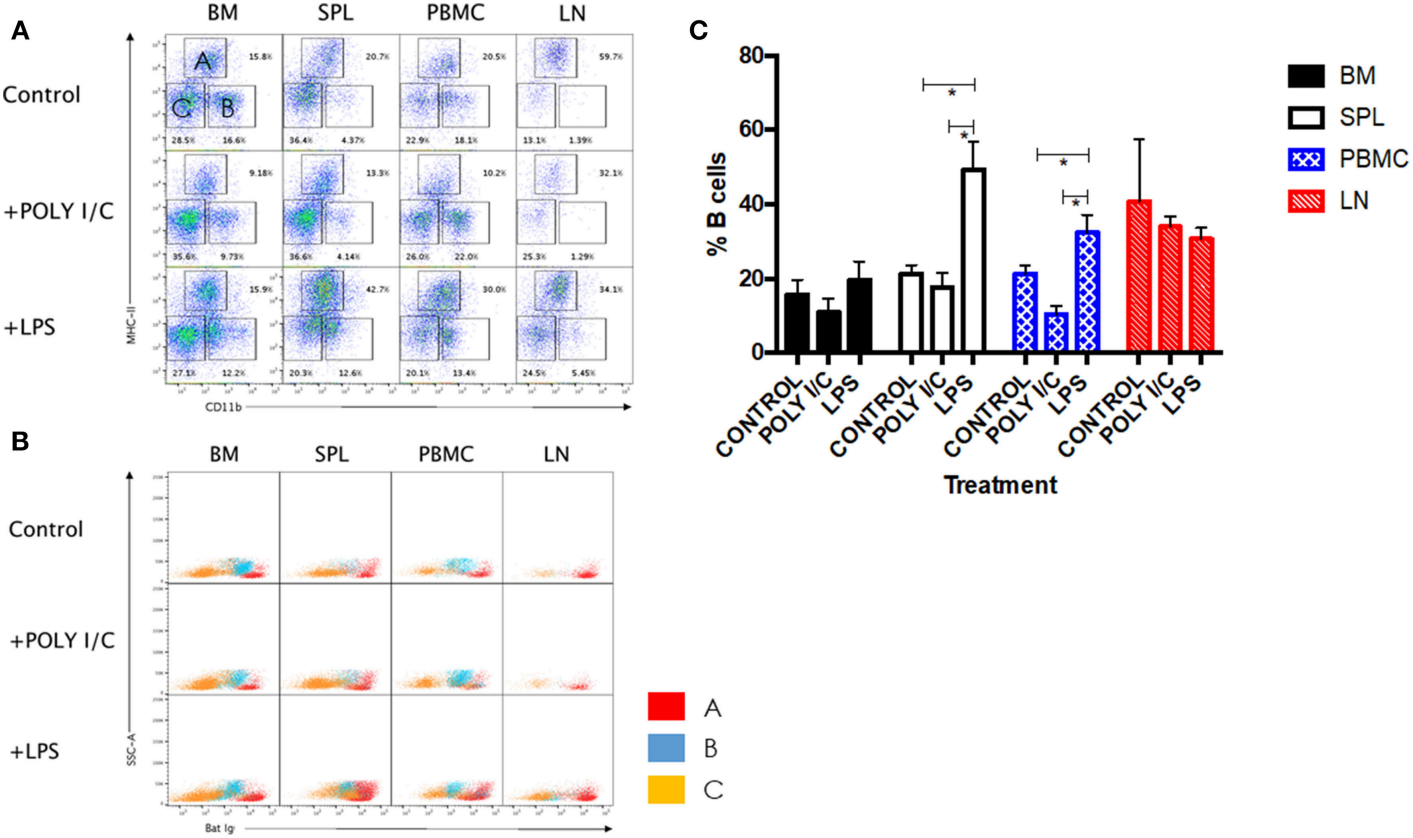
Analysis of immune cell populations in *P. alecto* treated with LPS or Poly I:C. **(A)** Cells from the bone marrow (BM), spleen (SPL), blood (PBMC) and lymph nodes (LN) were harvested 5 h after sc. injection with either LPS or Poly I:C, and analyzed by flow cytometry. Lymphocytes were gated based on forward and side scatter distribution. Three distinct populations were clearly obtained based on CD11b and MHC-II staining, namely subset A (CD11b^+^/^−^ MHC-II^+^), subset B (CD11b^+^ MHC-II^−^), and subset C (CD11b^−^ MHC-II^−^). Representative data for one bat is shown. **(B)** All three subsets were analyzed for Ig surface expression. **(C)** Percentages of B cells in BM, SPL, PBMC and LN from untreated, LPS treated and poly I:C treated (*n* = 3) bats 5 h post-treatment. Students *t*-test was used for the statistical analysis. ^*^p < 0.05.

LPS has long been known as both a TLR4 agonist and a T-independent antigen capable of inducing polyclonal B cell activation (28). We therefore sought to determine the ability of bat B cells to proliferate upon LPS stimulation. Groups of three wild-caught *P. alecto* bats were injected subcutaneously with LPS, poly I:C (a TLR3 agonist with no known B cell activation properties) or left untreated, and all nine animals were sacrificed 5 h later. Their bone marrow, spleen, lymph nodes (LN) and PBMC were harvested, single cells suspensions were prepared and subjected to flow cytometry analysis using the selected cross-reactive antibody panel. Lymphocytes were first gated based on forward and side scatter distribution. Three cell population subsets (A, B, and C) were then distinguished based on their expression of CD11b and MHC-II markers (Figure 5A). The CD11b^+/−^ MHC-II^+^ subset (A) contains mainly B cells (note that when compared against the FMO control, these cells are considered CD11b^−^); the CD11b^+^ MHC-II^−^ subset (B) contains myeloid cells; and the CD11b^−^ MHC-II^−^ subset (C) contains mainly T cells (confirmed by CD3^+^ staining; data not shown). These three subsets were further analyzed based on the detection of sIg. Consistently, the MHC-II^+^ CD11b^+/−^ cells (subset A) stained positive for sIg, whereas CD11b^−^ MHC-II^−^ cells (subset C) stained negative for sIg, and CD11b^+^ MHC-II^−^ cells (subset B) displayed an intermediate phenotype (Figure 5B). The latter observation may result from non-specific binding of antibodies to Fc receptors expressed at the surface of myeloid cells.

LPS-treated bats displayed a significant increase in the percentage of B cells in their spleens and PBMC compared to untreated control bats (Figure 5C). In contrast and expectedly, poly I:C treatment did not impact on the percentage of B cells. Poly I:C, a TLR3 agonist, is indeed not known to affect the adaptive immune cell populations (20). Absence of increased B cell population in LN and bone marrow is likely due to the short time treatment course (5 h) that did not allow the antigen to accumulate in these tissues in sufficient amount. No significant differences in the percentages of T and myeloid cells were observed in all organs and PBMC among the three groups (data not shown).

Overall, the data support that similar to other mammals, bat B cells activated and proliferated in response to stimulation with LPS, a T-independent antigen. Interestingly however, increase in bat B cell population could be observed as early as 5 h post-LPS treatment, while LPS-induced murine B cell *in vitro* proliferation is observed after several days of stimulation (29).

### BCR Cross-Linking Results in Calcium Influx

**Figure 6:**
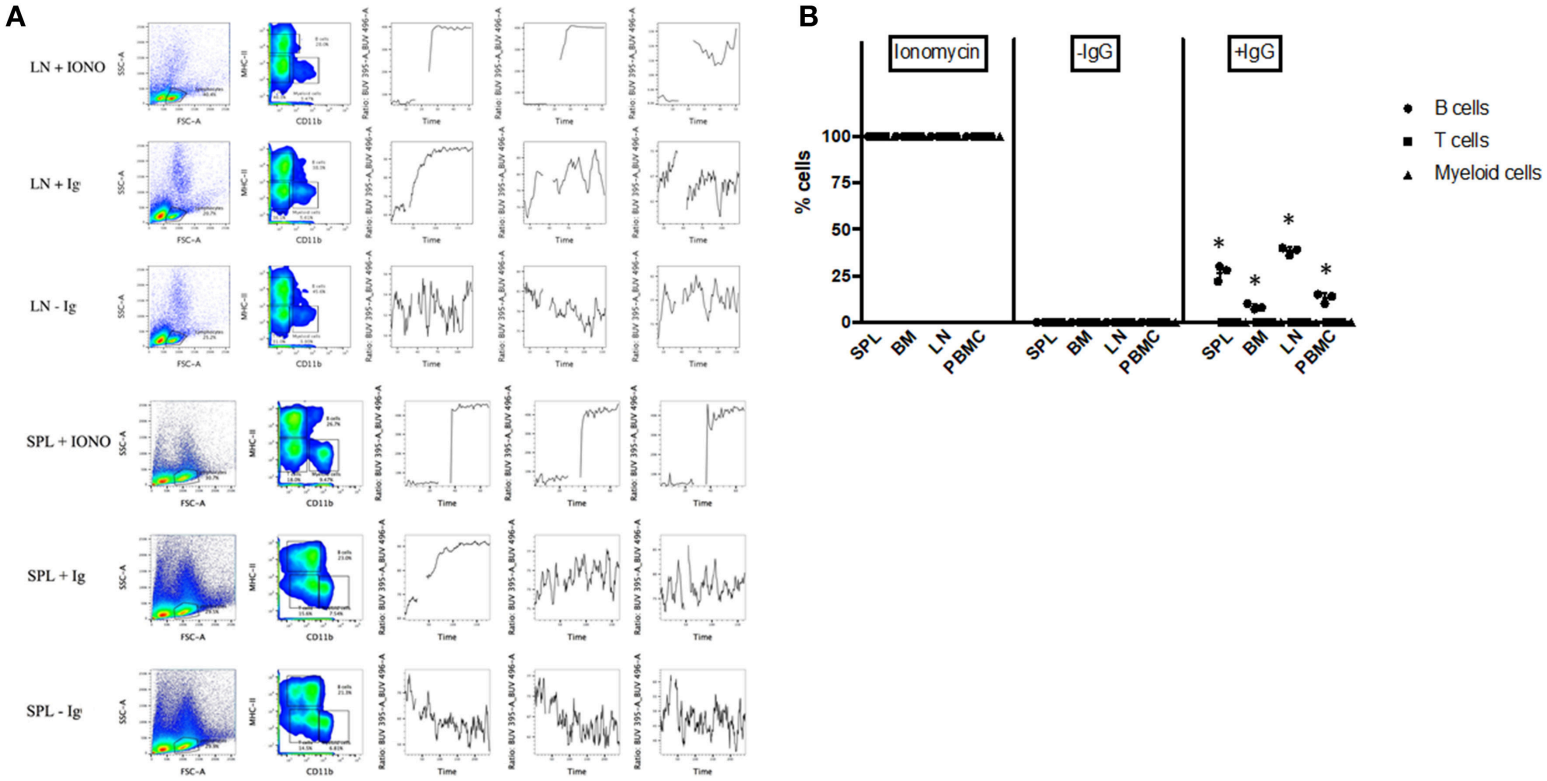
Calcium Release Assay. Bone marrow cells (BM), splenocytes (SPL), PBMC and lymph nodes (LN) from *P. alecto* bats (*n* = 3) were processed for calcium release assay as described in M&M. Cells treated with Ionomycin were used as positive control and cells stained without anti-bat Ig were used as negative control. **(A)** Lymphocytes were gated based on forward and side scatter distribution. B cells, T cells and myeloid cells were gated as CD11b^−^ MHC-II^+^, CD11b^−^ MHC-II^−^, and CD11b^+^ MHC-II^−^, respectively. One data set is shown and is representative of 3 independent experiments performed with 3 different bat spleens and lymph nodes. **(B)** Percentage of cells in BM, SPL, PBMC, and LN undergoing calcium influx after treatment with Ioniomycin, or with or without anti-IgG Ab. ^*^p < 0.05.

To further assess the functionality of bat B cells, a calcium release assay was conducted. Single cell suspensions prepared from *P. alecto* bone marrow (BM), spleen (SPL), PBMC and lymph nodes (LN) were incubated with a calcium sensor dye, Indo-1 which has a peak emission at 475 nm that shifts to 410 nm when bound to calcium. Indo-1 treated cells were then surface-stained with cross-reactive antibodies specific to MHC-II and CD11b, and with goat anti-bat Ig. The samples were also incubated with donkey anti-goat secondary antibody. Binding of the secondary antibody to goat anti-bat Ig primary antibody is expected to lead to B cell receptor (BCR) cross-linking (30) that results in Ca^2+^ entry inside the cells (31). Ca^2+^ influx inside Indo-1 pre-treated cells can be quantified at 410 nm over time. As described in the previous section, lymphocytes were gated based on forward and side scatter distribution. B cells, T cells and myeloid cells were gated as CD11b^−^ MHC-II^+^, CD11b^−^ MHC-II^−^, and CD11b^+^ MHC-II^−^, respectively. All three populations exhibited strong increase in calcium influx upon stimulation with Ionomycin (positive control) in all the samples tested (Figures 6A, B). Samples where anti-bat Ig antibody was omitted did not show any significant increase in calcium influx (negative control). Significant increase in calcium influx was observed with B cells but not T cells or myeloid cells, incubated with anti-bat Ig antibody (Figures 6A, B). The strongest signals were detected with B cells from SPL and LN (Figure 6B). This latter observation may be explained by the fact that these lymphoid organs harbor a greater proportion of mature B cells compared to the bone marrow, which is expected to contain predominantly progenitor, and immature B cells.

### Antibody Cross-Reactivity in *Eonycteris spelaea* Bats

**Figure 7:**
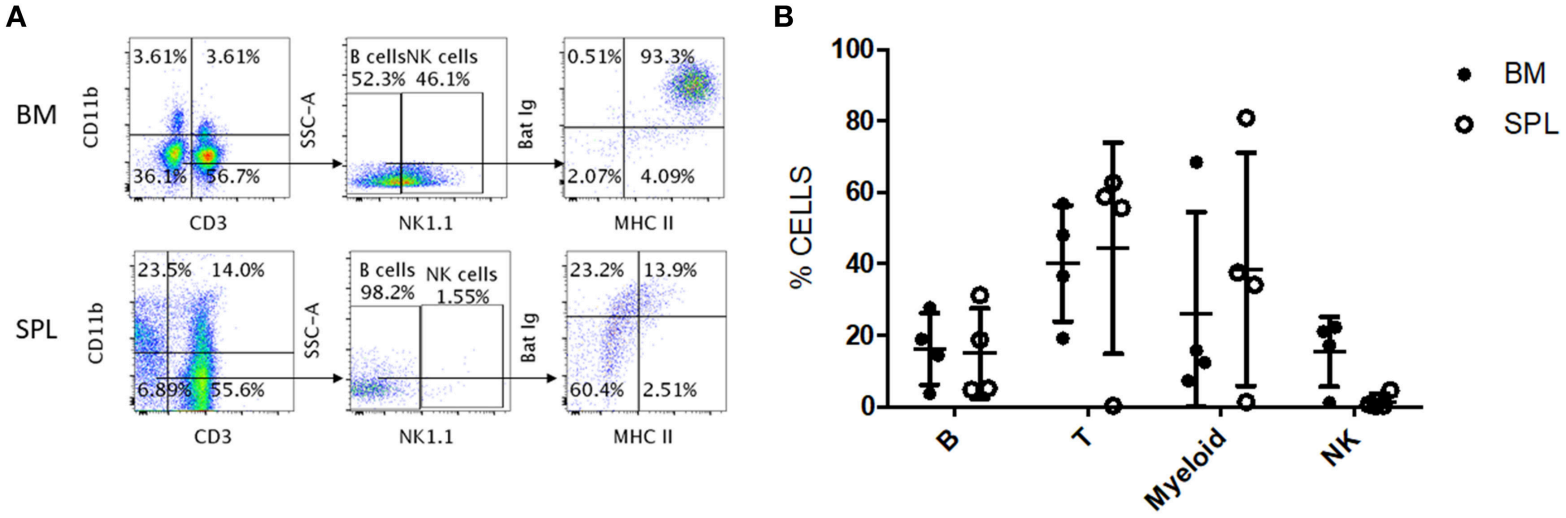
Flow cytometry analysis of *E. spelaea* immune cells using cross-reactive antibodies. **(A)** *E. spelaea* immune cells from spleen and bone marrow were analyzed by flow cytometry. CD3^+^ CD11b^−^ cells were identified as T cells and CD3^−^ CD11b^+^ cells as myeloid cells. CD3^−^ CD11b^−^ were further characterized based on NK1.1 to exclude NK cells and the CD3^−^ CD11b^−^ NK1.1^−^ cells were identified as B cells. The B cell population was further identified by detection of MHC-II and sIg. Gates were set using FMO controls. **(B)** Individual percentages of B, T, myeloid and NK cell populations in the bone marrow and spleen of *E. spelaea* bats. Percentages of cells from 4 individual bats (*n* = 4).

We tested whether the antibody panel identified for *P. alecto* would also be useful to study the adaptive immune cells from the fruit-eating cave nectar bat (*Eonycteris spelaea). E. spelaea* bats were caught from the wild in Singapore and were housed in a clean facility for several months before being euthanized for analysis of their immune cell populations. Housing these bats in a controlled and clean environment for several months is likely to bring the bats' immune system close to “steady state” or basal level compared to wild caught bats that are constantly exposed to a variety of unknown pathogens. The cross-reactive antibody panel that was selected for flow cytometry analysis of *P. alecto* samples, was applied on *E. spelaea* bone marrow and spleen samples. B, T, myeloid and NK cell subsets were clearly identified demonstrating that our antibody panel can be employed for flow cytometry analysis of adaptive immune cell populations in different bat species (Figure 7A). This finding thus implies a certain sequence conservation of adaptive immune cell surface markers amongst bat species.

Analysis of the percentages of immune cells revealed that *E. spelaea* colony bats harbor around 40–50% T cells in their spleen, similar to wild caught *P. alecto* bats (Figure 7B). Interestingly, and in sharp contrast to wild caught *P. alecto* bats, *E. spelaea* bone marrow was found to contain a large number of CD3^+^ T cells (~40 vs. <20% in *P. alecto*), and a much lower percentage of B cells (<20 vs. 60% in *P. alecto* bats). The percentages of NK cells in the bone marrow and spleen of *E. spelaea* were also rather different from those seen in *P. alecto* bats. Less than 10% of NK cells was indeed seen in the spleens from *E. spelaea* (Figure 7), vs. 10–16% in *P. alecto* (Figure 4). These discrepancies may be attributed either to inherent differences in the immune system between *E. spelaea* and *P. alecto* bats, and/or to the status of their immune system, since the former group had been housed in a controlled and clean environment for several months whereas the latter consists of wild caught animals.

## Discussion

This work confirms cross-reactivity of antibodies against surface molecules MHC-II, CD11b and intracellular protein CD3 on *P. alecto* cells as previously reported by us (22). Here we further extended the list of cross-reactive antibodies that are useful to study T, B, and NK cell populations. We also showed that these antibodies cross-reacted with immune cells from *E. spelaea*, a fruit-eating bat species within the genus *Eonycteris* from the megabat suborder, Megachiroptera, family of Pteropodidae that resides in many parts of Southeast Asia including Singapore and is not an endangered species (32). *E. spelaea* bats have been implicated in the 1999 Nipah virus outbreak in Malaysia, which led to the slaughter of one million pigs and caused 105 human deaths (33).

The successful identification of a panel of cross-reactive antibodies contributes significantly to the limited toolbox currently available to study bats adaptive immune system (34). Indeed, in contrast to innate immune cells, particularly DCs, that appear to be very conserved across mammals including bats (L.-F Wang, personal communication), the limited cross-reactivity observed in this study suggests that the adaptive immune cells in bats have diverged from other mammals such as humans and mice, which may point at significant functional differences.

The lack of proper tools to study bat species has hampered the progress of our understanding of the unique physiological attributes of bats (7, 13) and much of our knowledge has thus far been derived from transcriptomic and genomic studies (1, 14, 35–37). Although generating bat specific antibodies remains the best option, this approach is time consuming and expensive (22, 38, 39). Hence identifying cross-reactive antibodies allows for much quicker and cost-effective ways of studying bat immune cell populations. While identifying cross-reactive antibodies to study new species is a common approach (38, 40), new methods such as computational design strategies to improve the affinity of antibodies have been employed to increase species cross-reactivity of antibody candidates and could be exploited in the field of bat immunology (41).

The characterization of immune cell populations, including B cells, T cells and NK cells, in this study extends our previous study (22). Here, we have used cross-reactive NK1.1 antibody to identify the NK cells from CD3^−^ sIg^−^ gated population while we previously relied on the differential expression of transcription factors Tbet and Eomes to identify NK cells (22). Thus, antibody detection of surface molecule NK1.1 offers an improved approach for analysis of the NK cell population. Furthermore, the analysis of B cell population with cross-reactive antibodies against CD21 and CD27 has helped identify mature and memory B cell subsets, respectively, from the total B cell population identified as sIg^+^ CD3^−^.

While this study reported the percentage of T cell population in bat spleen to be more than 50%, which is similar to our previous findings (22), the percentage of T cells found in the bone marrow was about 10%, which is significantly <30% we previously reported. As for B cells, similar percentages of B cells in the spleen (close to 40%) were seen for both studies, however the B cell population in bone marrow from this study revealed a percentage close to 60 vs. 30% in our previous study. The discrepancies between both studies may stem from a different immune status between the two batches of wild caught bats, which were obtained from two different trips at different time of the year. Indeed, status of the immune system in these bats could be affected by past or ongoing infections, which may also be seasonal. Together with the limited sample size, variability in health status of wild caught bats inevitably leads to great variations among the data collected, thereby making difficult their interpretation. This underscores the need to establish bats colonies housed in a controlled and clean environment, allowing scientists to study the bat immune system at “steady state.” Making use of the recently established colony of *E. spelaea* bats, we analyzed the T and B cell content in their bone marrow and spleen. Data revealed striking differences in term of B, T and NK cell percentages in the bone marrow of *E. spelaea* compared to *P. alecto* bats, which may be explained either by inherent differences between both bat species and/or by a different immune status between colony bats and wild caught bats. Nevertheless, the consistent finding of a significantly higher percentage of T cells in bat spleen and PBMC compared to other mammals including mice and humans, strongly suggests that bats adaptive immune system has evolved to respond effectively to intracellular pathogens, which may explain their unique ability to cope with a large variety of viruses, hence acting as healthy reservoirs.

The predominance of T cells in bats does however not exclude a role for B cells. Our work suggests that bat B cells are functional and are capable of responding to LPS stimulation. LPS has been mainly known as a TLR4 agonist capable of triggering an innate inflammatory response in vertebrates (42, 43). LPS treatment studies have previously been carried out in pteropid bat cell lines (44) as well as in primary bat cells (8), and demonstrated the presence of a TLR4 response in bats. However, LPS is also a T cell-independent B cell stimulatory molecule, and as such is able to induce a polyclonal B cell proliferation response (28). Here, leveraging on this latter property, we found that LPS-treated bats displayed a greater proportion of B cells in their spleen and blood compared to untreated bats. It was rather surprising to detect an increase in the proportion of B cell population only 5 h post-LPS challenge, as typically LPS-induced B cell proliferation takes much longer (29). Wild caught bats are exposed to multiple bacterial pathogens. Therefore, it is plausible that the bat immune system, including the innate and adaptive components, has evolved to become particularly fast at responding to LPS. Furthermore, we showed that upon cross-linking of their BCR bat B cells responded by releasing Ca^2+^ from their ER stores, further supporting that bat B cells are fully functional.

In conclusion, this study has contributed to improve the current knowledge of bat adaptive immune cell populations. The identification of a panel of cross-reactive antibodies allows better characterization of the various adaptive immune cell populations in bats. In future, the field would greatly benefit from studies carried out in more controlled environments to allow analysis of the bat immune system at steady state. Besides offering new insights into the relationship between bats and viruses, the knowledge derived from bat immunology has the potential to be translated into clinical applications for humans, especially in the fields of virology and inflammatory response.

## Author Contributions

PP: designed experiments; PP, PH, JC, IB, SS, PS, and YH: performed experiments; AI, MB, and GC: provided materials; PP, JC, and SA: performed data analysis; C-AD, KF, and L-FW: provided intellectual inputs; SA and PP: wrote the paper.

### Conflict of Interest Statement

The authors declare that the research was conducted in the absence of any commercial or financial relationships that could be construed as a potential conflict of interest. The reviewer TS declared a past co-authorship with one of the authors, MB, to the handling editor.
