# Supplementary material for: Studies on B Cells in the Fruit-Eating Black Flying Fox (Pteropus alecto)
Source: Front Immunol. 2019 Mar 14;10:489. doi: 10.3389/fimmu.2019.00489 (PMC6428034; doi:10.3389/fimmu.2019.00489)
Supplement: Supplementary file 1 [file Data_Sheet_1.docx]

**Studies on B cells in the fruit-eating black FLYING FOX (*Pteropus alecto)***

**Supplemental Material**

**Authors:** Pravin Periasamy^1,2^, Paul E. Hutchinson^2^, Jinmiao Chen^3^, Isabelle Bonne^2^, Shahana Shereene D/O Shahul Hameed^1,2^, Pavithra Selvam^1,2^, Ying Ying Hey^4^, Katja Fink^3^, Aaron Trent Irving^4^, Charles-Antoine Dutertre^3,4^, Michelle Baker^5^, Gary Crameri^5^, Lin-Fa Wang^4^ and Sylvie Alonso^1,2*^

**Affiliations:** ^1^Department of Microbiology and Immunology, Yong Loo Lin School of Medicine, National University of Singapore, Singapore. ^2^Immunology programme, Life Sciences Institute, National University of Singapore, Singapore. ^3^Singapore Immunology Network (SIgN), Agency for Science, Technology and Research (A*STAR), Singapore. ^4^Programme in Emerging Infectious Disease, Duke-NUS Medical School, Singapore. ^5^CSIRO, Livestock Industries, Australian Animal Health Laboratory, Geelong, Australia.

*Corresponding author: Immunology programme, CeLS building, 28 Medical Drive, NUS, Singapore 117456. Tel: +65 65163541; Email: [micas@nus.edu.sg](mailto:micas@nus.edu.sg); Fax: +65 67782684.

**Table S1: Commercial antibodies raised in other species that were used to test the cross reactivity with *P. alecto* bats molecules**. Antibodies targeting immune cell markers from various species and clones with specific fluorochromes attached were tested. Fluorescein isothiocyanate (FITC), R-phycoerythrin (PE), Allophycocyanin (APC), BD Horizon V500 (V500), PE-Cy7 (Phycoerythrin-Cyanin 7).

| **List of Antibodies** | | | |
| --- | --- | --- | --- |
| **Name** | **Species** | **Clone** | **Fluorochrome** |
| CD43 (leukosialin) | Mouse | eBioR2160 | FITC Conjugated |
| CD135 | Mouse | A2F10 | PE |
| IgG1 | Mouse | MOPC-21 | APC |
| CD44 | Rat anti Mouse | IM7 | V500 |
| CD86 | Human | IT2.2 | Pacific Blue |
| CD21 | Mouse anti Human | B-Ly4 | BUV737 |
| CD4a | Mouse anti Pig | 74-12-4 | PerCP-Cy |
| CD11b | Mouse | M1/70 | PE |
| 1-Ad/1-Ed | Rat anti-Mouse | 2Gg | PE |
| Ig | Goat anti-bat Ig | polyclonal | NB7237 |
| Gata 3 | Human/Mouse | TWAJ | PerCP-Cy-eFluor710 |
| CD11b | Mouse | M1/70 | eFluor450 |
| CD45R | Human/Mouse | RA3-6B2 | APC |
| CD3 | Rat anti human | - | FITC |
| CD11b (integrin alpha M, Mac-1 alpha) | - | - | APC eFluor 780 |
| EOMES | Mouse | DanIIMag | eFluor 450 |
| EOMES | Mouse | DanIIMag | PE eFluor 610 |
| Tbet | Human/Mouse | eBio4B10 | PE - Cyanine 7 |
| Gata 3 | Human/Mouse | TWAJ | eFluor660 |
| Gata 3 | Human/Mouse | TWAJ | eFluor660 |
| IgG (H + L) | Goat anti Rat | - | Alexa Fluor 647 |
| 1L-10 | Human | JES3-19F1 | APC |
| IgG (H + L) | Donkey Anti Goat | - | Alexa Fluor 647 |
| IgG (H + L) | Donkey Anti Goat | - | Alexa Fluor 350 |
| CD56 | Human | MEM188 | FITC |
| BCl-6 | Mouse | BCL-DWN | PerCP-eFluor 710 |
| Tbet | Human/Mouse | eBio4B10 | PE-Cyanine 7 |
| CD8a | Human | HIT8a | PE |
| CD3 RM0027-3B19 | Rat | Monoclonal |  |
| CD19 | - | - | PE |
| CD8 | - | 32-M4 | PerCP |
| CD4 | - | RIV6 | FITC |
| CD3 | - | 5B2 | FITC |
| CD4 | - | MEM-115 | - |
| CD19 | - | eBio1D3 | PE Conjugated |
| CD19 (HIJ237) | Mouse | eBio1D3 | PE Conjugated |
| CD8 | - | UCH-T4 | PE |
| CD3 | - | KT3 | FITC |
| CD9 | Human | HI9a | PE |
| IgG1 | Rat anti Mouse | A85-1 | PE |
| CD9 | Human | HI9a | FITC |
| Blimp-1 | Rat anti Mouse | SE7 | BUV421 |
| GL7 | Human/Mouse | GL-7 | eFluor450 |
| F(ab')2 IgG | Mouse | polyclonal | PerCP-eFluor 710 |
| IgG2b, κ (IC) | Rat | - | PE Conjugated |
| LSEC | Mouse | - | FITC |
| RunX3 | Mouse | R3-5G4 | PE |
| CD154 (CD40L) | Human | 24-31 | PerCP-eFluor 710 |
| CD36 | Mouse | CRF D-2712 | APC |
| IgG2a (IC) | Rat | eBR2a | APC |
| CD4 | Mouse | RM4-5 | APC |
| CD155 | Mouse | TX56 | APC |
| CD40 | Mouse | IC10 | APC |
| ?CD18CFA | Mouse | ?2B1 | APC |
| MHC Class I | Mouse | ?SF1-11.1 | APC Conjugated |
| F(ab') IgG | Rat | polyclonal | APC |
| CD155 | Mouse | TX56 | APC Conjugated |
| ?CD3 | Mouse | 145-2C11 | APC Conjugated |
| CD36 | - | CRF D-2712 | APC |
| F(ab')2 IgG | Rat | Polyclonal | APC |
| CD49b (pan NK cells) | Mouse | DX5 | APC Conjugated |
| NK-1 | Mouse | PK136 | APC Conjugated |
| IgM | Mouse | 11141 | APC Conjugated |
| IgG1,κ (IC) | Mouse | P3 | APC Conjugated |
| CD45R | Mouse | RA3-6B2 | APC Efluor 647 Conjugated |
| MHC Class I (H-2Kb) | Mouse | AF6-88.5.5.3 | APC |
| CD107a | Mouse | ID4B | eFLUOR 660 |
| IgG2a | Rat | eBR2a | APC efluor 780 Conjugated |
| TIGIT | Mouse | GIGD7 | Alexa Fluor 647 |
| CD62L | Mouse | MEL-14 | APC Conjugated |
| CD31 | Rat anti-mouse | MEL-13.3 | APC |
| TLR-1 (CD281) | Mouse | eBioTR23 | Alexa Fluor 647 Conjugated |
| BrdU | - | - | APC |
| CD324 | - | DECMA-1 | Alexa Fluor 647 Conjugated |
| CD62L | Mouse | MEL-14 | APC Efluor 780 |
| CD86 | Mouse | GL-1 | APC/Cy7 |
| F4/80 | Mouse | BM8 | APC |
| CD3e | Mouse | 17A2 | APC |
| CD117 (c-kit) | Mouse | 2B8 | APC Conjugated |
| IgG2b κ (IC) | Rat | eB149/10H5 | eFLUOR 660 |
| IgG1,κ (IC) | Rat | eBRG1 | APC Conjugated |
| CD40L, gp39 | Mouse | MR1 | APC Conjugated |
| TBK1 (p5172) | Mouse | J133-587 | Alexa Fluor 647 |
| CD90.1 (Thy-1.1) | Rat/Mouse | HIS51 | APC Conjugated |
| CD44 | Mouse/Human | IM7 | APC Conjugated |
| CD135 | Rat anti-mouse | A2F10.1 | APC |
| IgG2b κ (IC) | - | eB149/10H5 | APC |
| CD11c | Mouse | N418 | APC Conjugated |
| CD314 (blocking) | Mouse | MI-6 | Alexa Fluor 647 Conjugated |
| BrdU | Mouse | 3D4 | Alexa Fluor 647 Conjugated |
| Cleaved PARP (ASP214) | Mouse | F21-852 | Alexa Fluor 647 Conjugated |
| ?IgM | Mouse | 1141 | APC Conjugated |
| CD86 | Mouse | GL1 | APC |
| IgG2b | Rat | eB149/10H5 | eFLUOR 660 |
| CD11c | Mouse | N419 | APC Conjugated |
| NKG2D/CD314 (blocking) | Mouse | MI-6 | Alexa Fluor 647 |
| IgG2b | Rat | eB149/10H5 | eFLUOR 660 Conjugated |
| CD8b | Mouse | H35-17.2 | APC eFLUOR 780 |
| IgG (IC) | Armenian Hamster | eBio299Arm | APC Conjugated |
| IgM | Mouse | 11/41 | APC Conjugated |
| SINFEKL/H-2 κ b | Mouse | eBio25-D1.16 | APC |
| Gata 3 | Hu/Mo | TWAJ | eFLUOR 660 |
| CD27 | Mouse, Human, Rat | LG.7F9 | APC Conjugated |
| IgG2a κ (IC) | Mouse | MOPC-173 | Alexa Fluor 647 Conjugated |
| CD69 | Mouse | H1.2F3 | APC |
| IgG1,κ (IC) | Mouse | P3 | APC Conjugated |
| CD45R | Mouse/Human | RA3-6B2 | Alexa Fluor 647 Conjugated |
| CD5 (Ly-1) | Mouse | 53-7.3 | APC Conjugated |
| Ly6G and Ly6L (RB6-8C5) | Rat anti-mouse | - | APC Conjugated |
| IgM | Mouse | 11/41 | APC |
| Phospho-histone H2A.X (Set 139) 20E3 | Rabbit | - | Alexa Fluor 647 Conjugated |
| CD154 (CD40L, gp39) | Mouse | MR1 | APC Conjugated |
| TNF-α | Mouse | MP6-XT22 | APC Conjugated |
| CD62L | Mouse | MEL-14 | APC |
| CD278 (ICOS) | Human/Mouse/Rat | C398.4A | APC |
| IgA κ (IC) | Mouse | M18-254 | APC |
| Annexin V |  |  | APC |
| IgG2a κ (IC) | Mouse | eBM2a | Alexa Fluor 647 |
| IgG2a κ (IC) | Mouse | eBM2a | APC Conjugated |
| CD45R (B220) | Hu/Mo | RA3-6B2 | APC |
| IgG (IC) | Rabbit | - | Alexa Fluor 647 Conjugated |
| STAT3 (PY70S) | Mouse | - | Alexa Fluor 647 Conjugated |
| CD85? (1.Y-3) | Mouse | eBioH35-172 | APC Conjugated |
| IFN-γ | Mouse | XMG1.2 | APC Conjugated |
| IgG2b (IC) | Mouse | eBMG2b | Alexa Fluor 647 Conjugated |
| Ly6G and Ly6L (RB6-8C5) | Rat anti-mouse | - | APC |
| IgG2a κ (IC) | Rat | eBR2b? | Alexa Fluor 647 |
| CD63 | Mouse | NVG-2 | APC |
| CD48 (BCM1) | Mouse | HM48-1 | APC Conjugated |
| IgG2a | - | Monoclonal A20(A-12) | APC |
| CD117 (c-kit) | Mouse | ACK2 | APC Fluor 780 Conjugated |
| MHC Class 1 (H-2κ) | Mouse | AF6-88.5.5.3 | APC Conjugated |
| TBK1 (p5172) | Mouse | J133-587 | Alexa Fluor 647 |
| CD44 | Human/Mouse | IM7 | PE-Cyanine 7 |
| F4/80 | Mouse | BM8 | PE-Cy 7 |
| IgG2a κ (IC) | Rat | - | PE-Cy 7 |
| NK1.1 | Mouse | PK136 | PE Cy 7 |
| IgG1 (IC) | Rat | eBRG1 | PE CY 7 Conjugated |
| TER-119(Ly-76) | Mouse | TER-119 | PE Cy 7 |
| TER-119/Erythroid cells | Rat anti-mouse | TER-119 | PE Cy 7 |
| IgG (IC) | Golden Syrian Hamster |  | PE Cy7 Conjugated |
| IgM | Mouse | 11/41 | PE Cy7 |
| CD314 (NKG2D) | Mouse | CX5 | PE Cy7 |
| CD117 (c-kit) | Mouse | ACK3 | PE Cy 7 |
| CD28 | Mouse | 37.51 | PE Cy7 Conjugated |
| CD23 (FCER11) | Mouse | B3B4 | PE Cy7 Conjugated |
| CD45R (B220) | Human/Mouse | RA3-6B2 | PE CY 7 |
| CD127 (IL-7Ra) | Mouse | A7R34 | PE Cy7 Conjugated |
| IgG2a κ (IC) | Mouse | eBM2a | APC eFLUOR 780 |
| Ki-67 | Human | 20Raj1 | eFluor 660 |
| Ph2ax (5139) | Human/Mouse | CR55T33 | eFluor 660 |
| CD3e | Mouse | C7A2 | APC eFluor 780 |
| CD90.1 (Thy-1.1) | Mouse/Rat | HIS51 | APC eFluor 780 |
| IgG 2a (IC) | Rat | eBR2a | APC Alexa Fluor 750 Conjugated |
| CD8a | Mouse | 53-6.7 | APC eFluor 780 |
| pan NK Cells (CD49b) | Mouse | DX5 | APC Conjugated |
| CD8a | Mouse | 53-6.7 | APC eFluor 780 |
| IgG | Armenian Hamster | eBio299Arm | FITC Conjugated |
| IgG (IC) | Armenian Hamster | eBio299Arm | FITC Conjugated |
| BrdU | - | BU-1 | Alexa Fluor 488 Conjugated |
| IgG 2a (IC) | Rat | eBR2a | FITC Conjugated |
| IgG (IC) | Rabbit | - | FITC Conjugated |
| IgG2b (IC) | Rat | - | FITC Conjugated |
| IgM (IC) | Rat | eBRM | FITC |
| CD43 | Mouse | eBioR2/60 | FITC |
| IgG1 κ (IC) | Mouse | P3 | FITC Conjugated |
| IgG2a | Mouse | eBM2a | FITC Conjugated |
| IgG2b (IC) | Mouse | eBMG2b | Alexa Fluoro 488 Conjugated |
| CD4 (L3T4?) | Mouse | GK1.5 | FITC Conjugated |
| CD4 | Mouse | GK1.5 | FITC |
| IgG2α κ (IC) | Mouse | eBM2a | FITC |
| CD45 | Mouse | 30-F11 | FITC |
| CD8b | Mouse | eBioH35-17.2 | FITC |
| T cell receptor | Mouse | MGg-4 | FITC |
| CD45R | Human/Mouse | RA3-6B2 | FITC |
| IgG2a κ (IC) | Rat | eBR2a | FITC |
| CD107a (ID4B) | Rat, Mouse | - | FITC |
| CD69 | Mouse | HI-2F3 | FITC Conjugated |
| IFN-γ | Mouse | XMG1.2 | FITC Conjugated |
| Ki-67 | Mouse/Rat | So1A15 | FITC |
| CD282 (TLR) | Mouse | 6C2 | FITC |
| IL-1a | Mouse | ALF-161 | FITC Conjugated |
| Ly-49C | Mouse | *5E6 | FITC |
| Ly-6AJE (5ca-1) | Mouse | D7 | FITC Conjugated |
| IgM | Mouse | Eb121-15F9 | FITC |
| IgD | Mouse | 11.26 | FITC Conjugated |
| HLA - ABC | Human | W6/32 | Alexa Fluor 488 |
| MHC Class 1 | Mouse | 28-14-8 | FITC Conjugated |
| BrdU | - | PRB-1 | FITC Conjugated |
| TBK1 (p5172) | - | J133-587 | Alexa Fluor 488 |
| IL-4 | Mouse | 11B11 | PE |
| NK-1.1 | Mouse | PK136 | PE Conjugated |
| PDCA-1 (CD317) | Mouse | eBio927 | PE Conjugated |
| IgG2a (IC) | Rat | eBR2a | PE Conjugated |
| CD140b (PDGF2b) | Mouse | APB5 | PE Conjugated |
| CD150 | Mouse | MShad150 | PE |
| CD152 (CTLA-4) | Mouse | UC10-4B9 | PE Conjugated |
| CD121a | Rat anti-mouse | 35FC | PE |
| CD155 | Rat | TX21 | PE |
| CD155 (PVR) | Mouse | TX56 | PE |
| IL-6 | Mouse | 12-7061.81 | PE |
| CD112 | Human | TX31 | PE |
| CD226 (DNAM-1) | Mouse | 480.1 | PE |
| CD107a | Mouse | eBio1D48 | PE |
| CD126 (1E6Ra) | Mouse | D7715A7 | PE Conjugated |
| CD121a | Rat anti-mouse | 35FS | PE |
| CD126 (IL-6Ra) | Mouse | 12-129-80 | PE Conjugated |
| CD155 | Human | 2H7CD155 | PE |
| CD140a (PDGF2Ra) | Mouse | APA5 | PE Conjugated |
| CD8c | Mouse | 16-10A1 | PE |
| CD80 (B7-1) | Mouse | 16-10A1 | PE Conjugated |
| CD84 | Mouse | MCD84.7 | PE |
| CD127 (IL-7Ra) | Mouse | A7R34 | PE Conjugated |
| CD95 | Hamster anti mouse | - | PE |
| CD61 | Mouse | 2C9.G3 | PE Conjugated |
| CD62L | Mouse | MEL-14 | PE |
| CD70 | Mouse | FR70 | PE Conjugated |
| CD48 | Mouse | HM48-1 | PE |
| CD49b (Integrin α2) | Mouse | DX5 | PE |
| CD46 | Human | *8E2 | PE |
| CD155 | Human | 2H7CD155 | PE |
| CD11b | Mouse | M1/70 | PE Conjugated |
| CD4 | Mouse | RM4-4 | PE |
| CD11c | Mouse | N418 | PE |
| CD40 | Human | 5C3 | PE Conjugated |
| CD25 | Mouse | PC61.5 | PE |
| CD25 (IL-2Ra,p55) | Mouse | PC61.5 | PE |
| CD4 | Mouse | RM4-4 | PE |
| CD27 | Mouse, Human, Rat | LG.7F9 | PE Conjugated |
| IFNARI | Mouse | MAR1-5A3 | PE |
| F4/80 | Mouse | BM8 | PE |
| CD3e | Mouse | 145-2C11 | PE |
| CD3e | Mouse | 145-2C11 | PE Conjugated |
| T-bet | Human/mouse | eBio4B10 | PE |
| CD3e | Mouse | 145-2C11 | PE Conjugated |
| CD90.1 (Thy-1.1) | Mouse | ?-155 | PE Conjugated |
| CD18 | Mouse | M18/2 | PE Conjugated |
| CD29 (a4b1,VLA-4) | Mouse | eBioHMb 1-1 | PE Conjugated |
| IgG (IC) | Armenian Hamster | eBio299Arm | PE Conjugated |
| IgG 1 κ (IC) | Mouse | MOPC-21 | PE |
| CD19 | Mouse | eBio1D3 | PE Conjugated |
| CD86 (B7-2) | Mouse | P03.1 | PE Conjugated |
| IgG1 (IC) | Rat | eBRG1 | PE |
| IgG1,κ (IC) | Mouse | P3 | PE Conjugated |
| IgG2a κ (IC) | Mouse | eBM2a | PE Conjugated |
| IgG2b κ (IC) | Mouse | eBMG2a | PE |
| IgG1,κ (IC) | Rat | eBRG1 | PE |
| IgG1,κ (IC) | Mouse | P3 | PE Conjugated |
| IgG2b κ (IC) | Rat | Eb149/10H5 | PE |
| Ly-6G (GR-1) | Mouse | RB6 - 8C5 | PE Conjugated |
| γδ - TCR | Mouse | eBioGL3 | PE |
| γδ - TCR | Mouse | eBioGL3 | PE Conjugated |
| Ly G29K (IC) | Mouse | eBM2a | PE |
| CD45R (B220) | Human/mouse | RA3-6B2 | PE |
| IgG | F(ab')2 donkey anti mouse | Polyclonal | PE |
| IgM | Mouse | 11/41 | PE Conjugated |
| IgM | Mouse | Eb121-15F9 | PE Conjugated |
| CD281 (TLR1) | Mouse | eBioTR23 | PE |
| IL-6 | Mouse | MP5-20F3 | PE |
| MHC Class II (1-Aft-E) | Mouse | M51114.152 | PE Conjugated |
| Interlukin-6 | Mouse | MP5-20F3 | PE Conjugated |
| Ly 6G | Mouse | 1A8 | PE |
| Early B Lineage | Rat anti-mouse | AA4.1 | PE |
| NK-1.1 (NKR-PIC, ?Ly 55) | Mouse | PK136 | PE Conjugated |
| CD314 | Mouse | A10 | PE |
| CXCR5 | Rat anti-mouse | - | PE |
| MHC Class 1 (H-2κb) | Mouse | AF6-88.5.5.3 | PE Conjugated |
| BrdU | Mouse | Bu20a | PE |
| Integrin B7 | Mouse/Human | FIB504 | PE Conjugated |
| Ly6-A5E | Mouse | D7 | PE Conjugated |
| IL-17A | Mouse | - | PE |
| NKG2D | Mouse | C7 | FITC |
| IgG2b (IC) | Rat | eB149/10H5 | FITC Conjugated |
| CD4 | Human | OKT4 | FITC |
| CD11b | Mouse | M1/70 | FITC Conjugated |
| Ki-67 | Mouse/Rat | So1A15 | FITC |
| F4/80 | Mouse | BM8 | alexa Fluor 488 |
| CD34 | Mouse | RAM34 | FITC Conjugated |
| Prominin 1 (CD133, AC133) | Mouse | 13A4 | FITC Conjugated |
| CD90.1 (Thy-1.1) | Rat/Mouse | HIS51 | FITC Conjugated |
| CD45R (B220) | Human/Mouse | RA3-6B2 | FITC |
| CD127 (IL-7Ra) | Mouse | ALR34 | FITC Conjugated |
| Nectin-2 | Mouse | 502.57.1 | FITC |
| CD11c | Mouse | N418 | FITC Conjugated |
| CD86 | Mouse | GL1 | FITC |
| CD117 (c-kit) | Mouse | 2B8 | FITC Conjugated |
| CD54 (ICAM-1) | Mouse | YN1/17.4 | FITC |
| CD11a (LFA-1alpha) | Mouse | M17/4 | FITC Conjugated |
| NK-1.1 (NKR-PIC, Ly 55) | Mouse | PK136 | FITC Conjugated |
| CD36 (B7.2)? | Mouse | GL1 | FITC Conjugated |
| pan NK Cells (CD49b) | Mouse | DX5 | FITC Conjugated |
| CD1d (CD1.1, Ly-38) | Mouse | IB1 | Alexa Fluor 488 Conjugated |
| CD144 | Mouse | eBioBr13 | Alexa Fluor 488 Conjugated |
| CD45R (B220) | Human, Mouse | RA3-6B2 | Alexa Fluor 488 Conjugated |
| IgG1,κ (IC) | Mouse | P3 | Alexa Fluor 488 Conjugated |
| IgG2b (IC) | Mouse | eBMG2b | Pacific Blue Conjugated |
| IgG1,κ (IC) | Mouse | P3 | Pacific Blue Conjugated |
| IgG2b (IC) | Rat | RTK4530 | Pacific Blue |
| IgG2b κ (IC) | Rat | eB149/10H5 | Pacific Blue Conjugated |
| IgG (IC) | Armenian Hamster | eBio299Arm | eFluor 450 Conjugated |
| IgG (IC) | Golden Syrian Hamster | - | Pacific Blue Conjugated |
| IgG2b κ (IC) | Rat | eB149/10H5 | eFluor 450 |
| IgG2b κ (IC) | Rat | RTK4530 | Brilliant violet 650 |
| CD8a | Mouse | 53-6.7 | eFluor 450 |
| CD8a (Ly-2) | Mouse | 53-6.7 | eFluor 450 Conjugated |
| Ly-6G (GR-1) | Mouse | RB6 - 8C5 | Pacific Blue Conjugated |
| CD3e | Mouse | 145-2C11 | eFluor 450 |
| IgG2a (IC) | Rat | eBR2a | eFluor 450 |
| Ly-6G (GR-1) | Mouse | RB6 - 8C5 | eFluor 450 |
| CD335 (NKP46) | Mouse | 29A1.4 | eFluor 450 |
| CD8a | Mouse | 53-6.7 | APC eFluor 780 |
| CD3 | Mouse | 17A2 | Brilliant violet 650 |
| Ly-6G (GR-1) | Mouse | RB6 - 8C5 | APC eFluor 780 |
| IgG2b κ | Rat | RTK4530 | Pacific Blue |
| CD44 | Human, Mouse | IM7 | eFluor 450 |
| CD54 | Mouse | YN1/17.4 | Pacific Blue |
| CD44 (P5P-T,Ly24) | Mouse/Human | IM7 | Pacific Blue Conjugated |
| IgG2b κ (IC) | Rat | eB149/10H5 | eFluor 450 |
| CD3 | Human | UCHT1 | Pacific Blue Conjugated |
| CD11b | Mouse | M1/70 | eFluor 450 |
| CD8a | Mouse | - | - |
| CD122 | Mouse | TM-b1 | eFluor 450 |
| Vα2 TCR | Rat anti-mouse | B20.1 | Per CP-Cy 5.5 |
| TCRβ | Mouse | H57-597 | Per CP-Cy 5.5 |
| Ly6A/E (sca-1) | Mouse | D7 | Per CP-Cy 5.5 |
| CD45R (B220) | Human/Mouse | RA3-6B2 | Per CP-Cy 5.5 |
| FOX p3 | Mouse/Rat | FJK-16a | Per CP-Cy 5.5 |
| NK1.1 | Mouse | PK136 | Per CP-Cy 5.5 |
| CD69 | Mouse | H1.2F3 | Per CP-Cy 5.5 |
| CD80 | Mouse | 16-10A1 | Per CP-Cy 5.5 |
| CD107a (LAMP-1) | Human | H4A3 | Alexa Fluor 647 |
| CD43 | Mouse | IB11 | Per CP-Cy 5.5 |
| IgG (IC) | Armenian Hamster | eBio299Arm | Per CP-Cy 5.5 Conjugated |
| CD3e | Mouse | 145-2C11 | Per CP-Cy 5.5 |
| CD8a | Mouse | 53-6.7 | Per CP-Cy 5.5 |


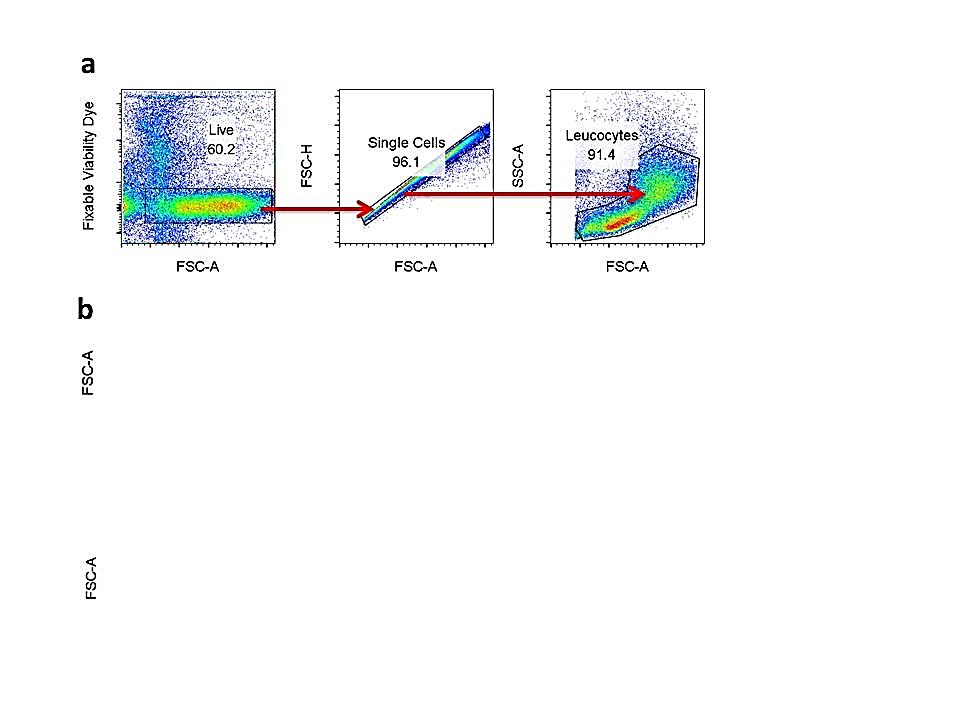

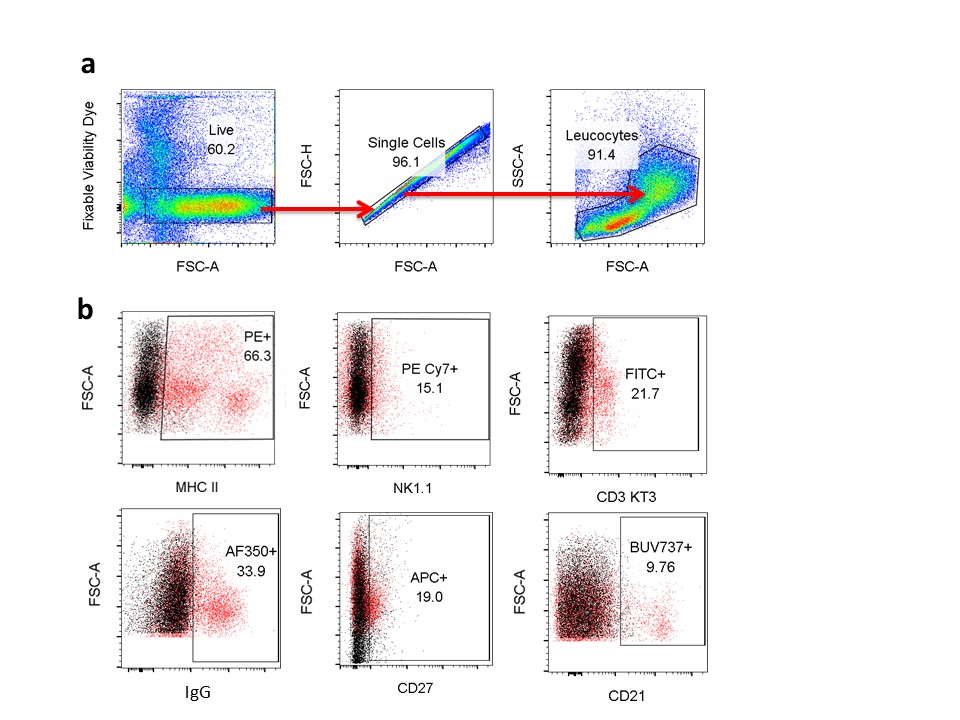


**c**


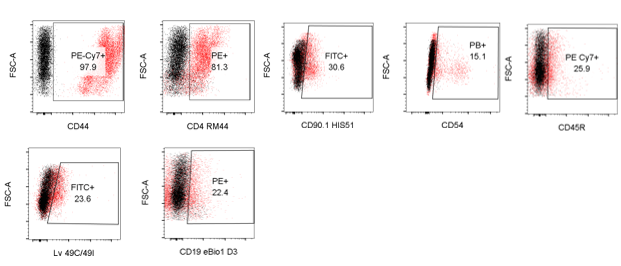


**d**


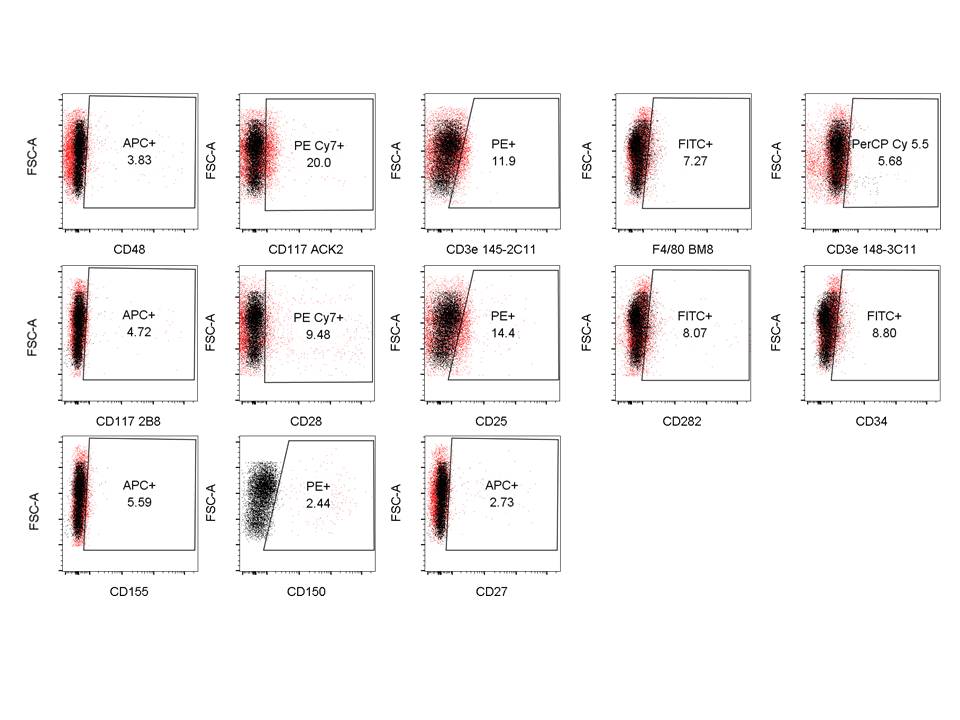


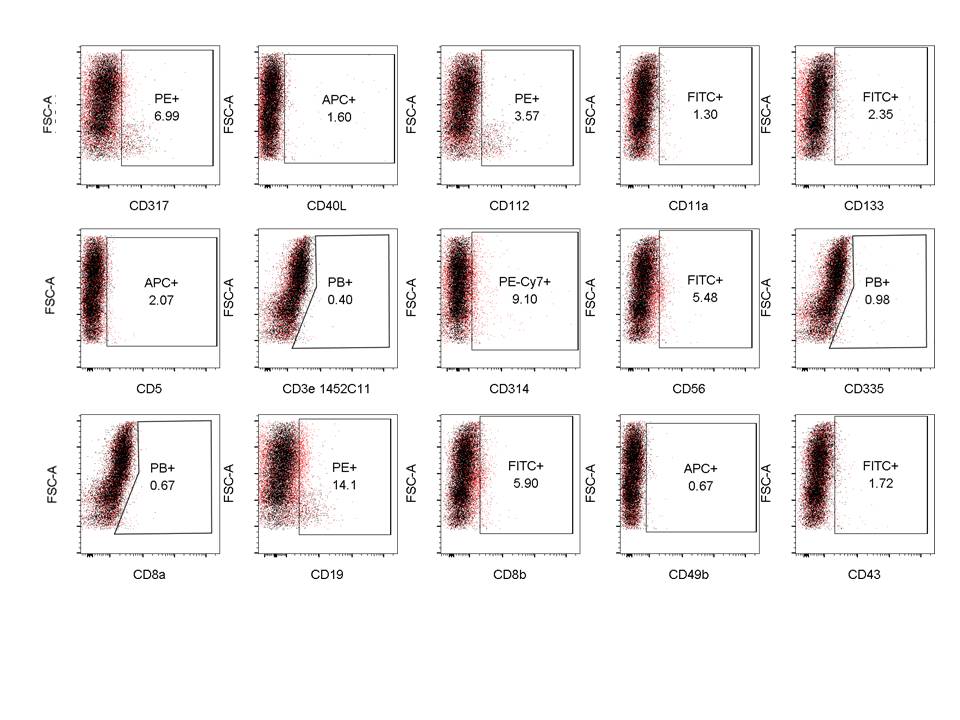

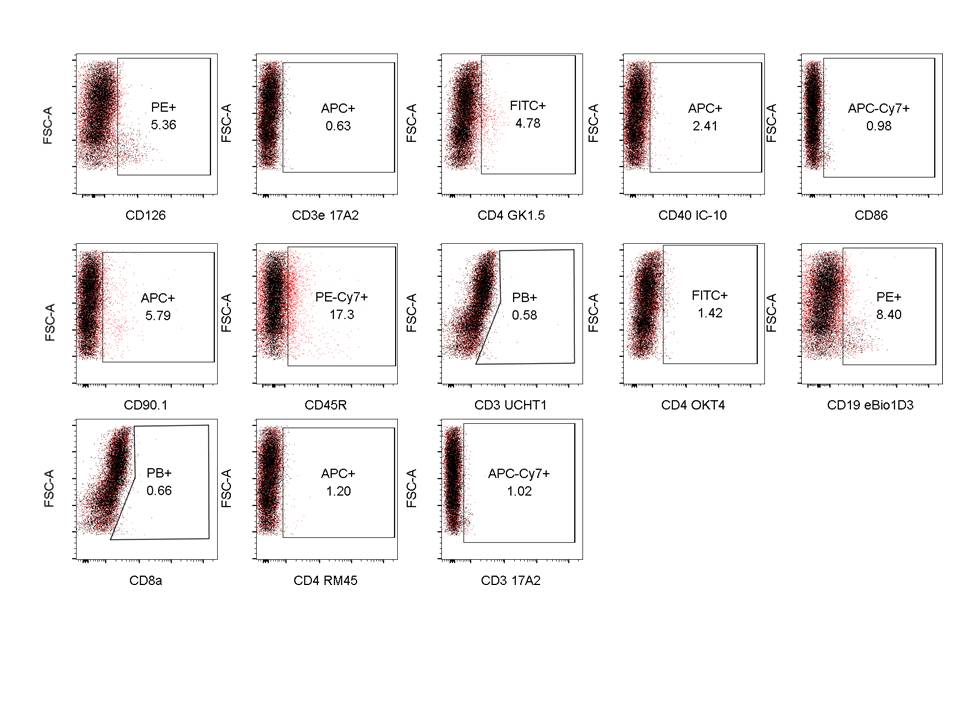

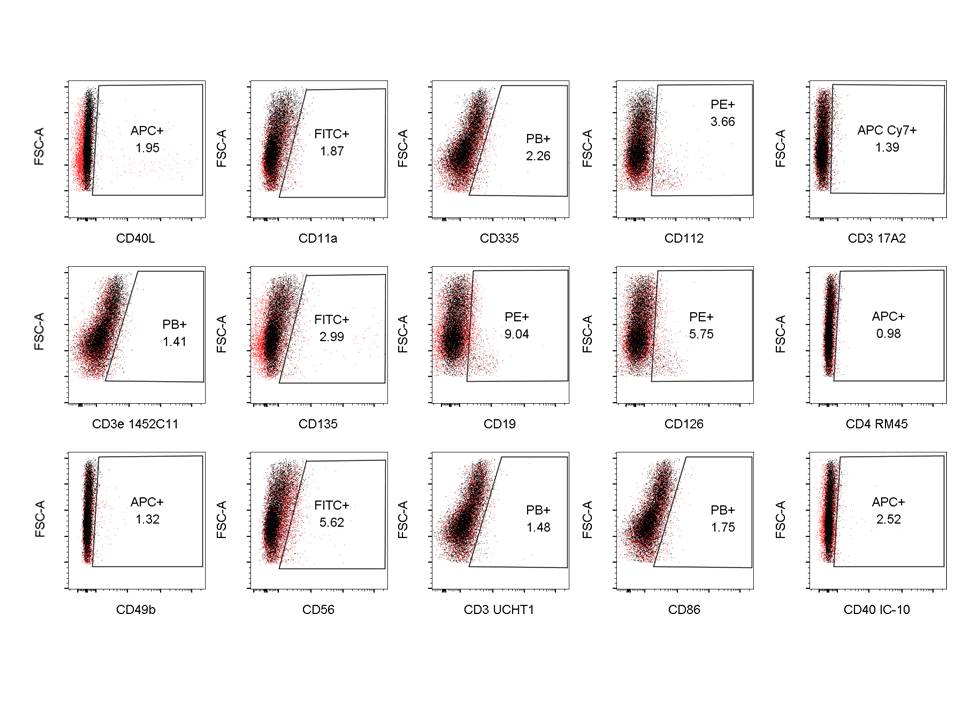

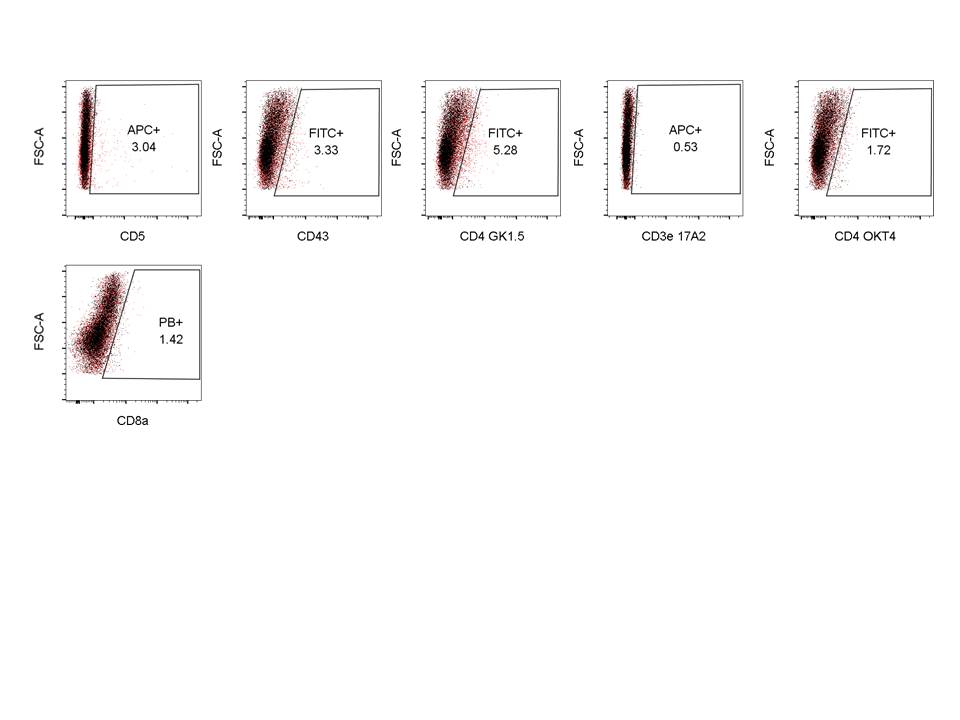

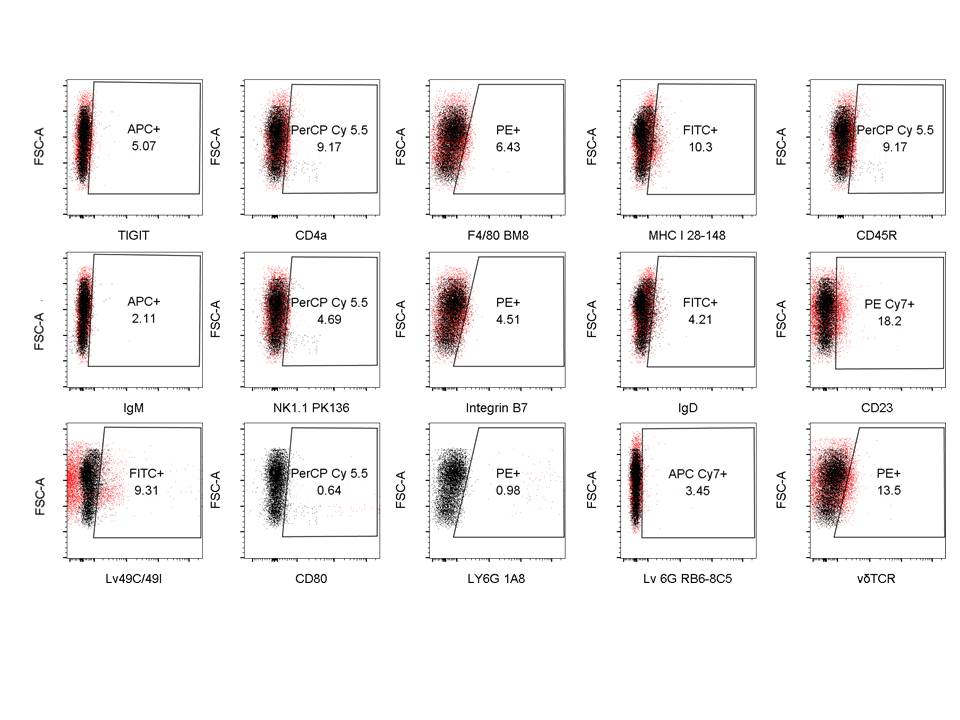

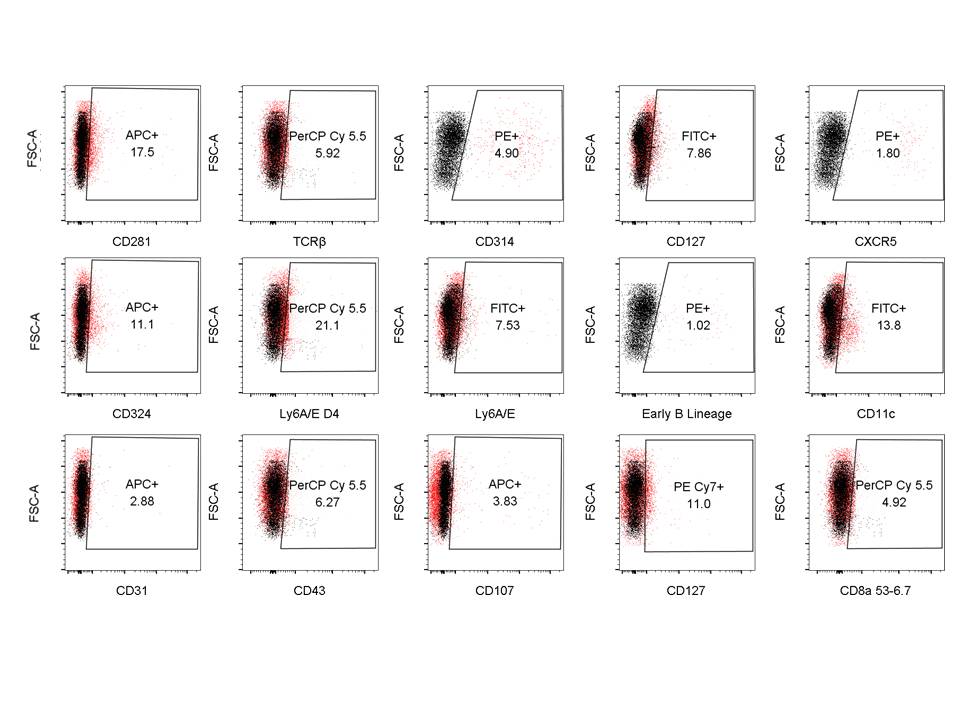

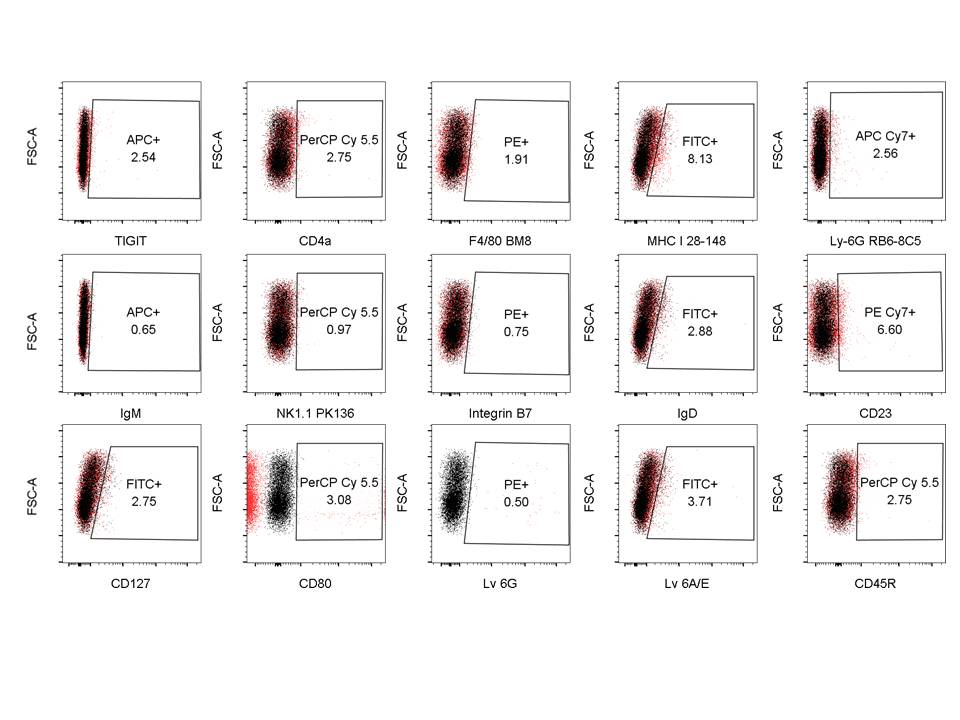

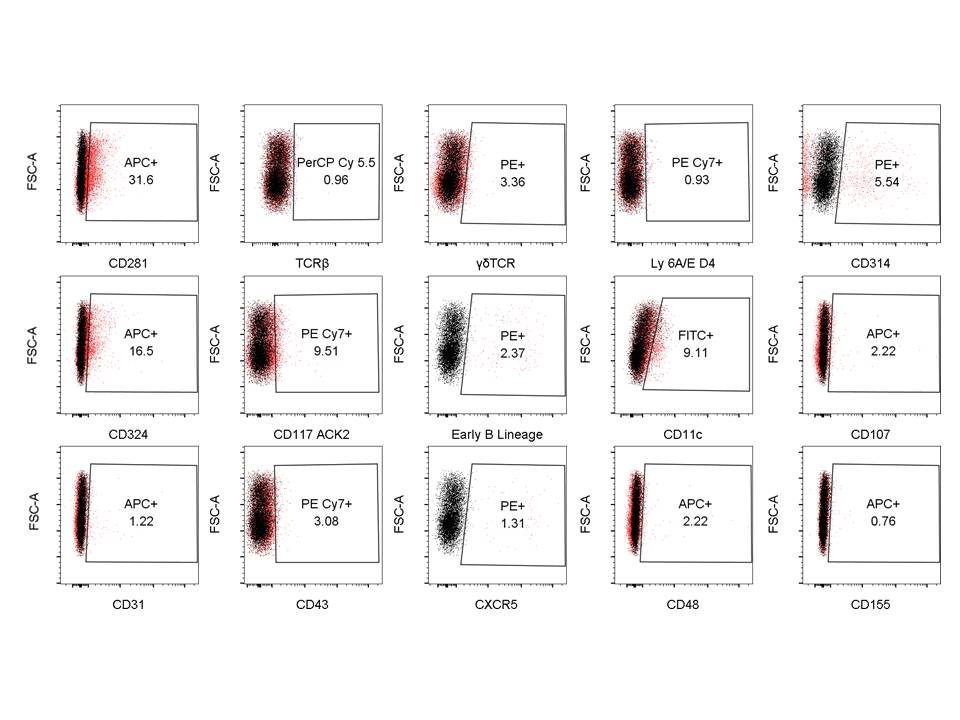

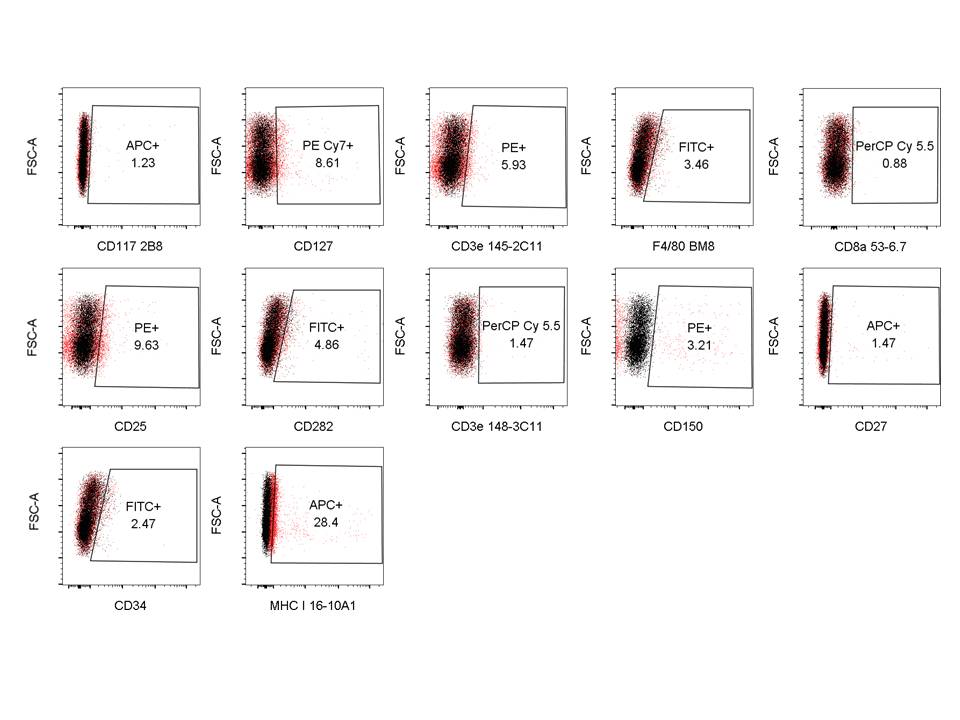


**Figure S1. Flow cytometry analysis of bat bone marrow and splenocytes using commercial antibodies.** 83 antibodies were tested individually. (a) Gating strategy to identify leucocytes; (b) Antibodies (n=6) that showed staining above background and were used in this study; (c) Antibodies (n=7) that showed staining above background but were not used in this study; and (d) Antibodies (n=68) that did not show significant staining above background and/or that did not lead to identification of a distinct cell population.
